# Obstructive Sleep Apnea and Arrhythmia: A Narrative Review of Arrhythmogenic Mechanisms

**DOI:** 10.3390/diagnostics16121885

**Published:** 2026-06-17

**Authors:** Crina Veronica Zinveliu (Bercian), Adela Viviana Sitar-Taut, Angela Cozma, Elena Buzdugan, Olga Hilda Orasan, Roxana Liana Lucaciu, Adriana Corina Hangan, Lucia Maria Procopciuc

**Affiliations:** 1 Department of Cardiology, “Dr. Constantin Papilian” Emergency Military Hospital, 400132 Cluj-Napoca, Romania; crina.bercian@gmail.com; 2 Doctoral School, “Iuliu Hațieganu” University of Medicine and Pharmacy, 400132 Cluj-Napoca, Romania; 3 4th Department of Internal Medicine, Faculty of Medicine, “Iuliu Hațieganu” University of Medicine and Pharmacy, 400132 Cluj-Napoca, Romania; angelacozma@umfcluj.ro (A.C.); hilda.orasan@umfcluj.ro (O.H.O.); 4 Department of Internal Medicine, 5th Medical Clinic, Faculty of Medicine, “Iuliu Hațieganu” University of Medicine and Pharmacy, Cluj Municipal Hospital, 400132 Cluj-Napoca, Romania; cristina.buzdugan@umfcluj.ro; 5 Department of Pharmaceutical Biochemistry and Clinical Laboratory, Faculty of Pharmacy, “Iuliu-Hațieganu” University of Medicine and Pharmacy, 400132 Cluj-Napoca, Romania; liana.lucaciu@umfcluj.ro; 6 Department of Inorganic Chemistry, Faculty of Pharmacy, “Iuliu Hațieganu” University of Medicine and Pharmacy, 400132 Cluj-Napoca, Romania; adriana.hangan@umfcluj.ro; 7 Department of Medical Biochemistry, Faculty of Medicine, “Iuliu Hațieganu” University of Medicine and Pharmacy, 400132 Cluj-Napoca, Romania; lprocopciuc@umfcluj.ro

**Keywords:** obstructive sleep apnea, sleep-disordered breathing, arrhythmia, arrhythmogenic mechanisms, ventricular arrhythmia, premature atrial contraction, atrial fibrillation

## Abstract

Obstructive sleep apnea (OSA) constitutes a chronic disorder characterized by recurrent upper airway collapse during sleep. This condition is prevalent among patients with cardiac rhythm disturbances and represents a potent independent risk factor for arrhythmia. Although most studies have concentrated on the association between OSA and atrial fibrillation (AF), numerous investigations have established connections with ventricular and supraventricular arrhythmias. Arrhythmogenesis in OSA represents a complex multifactorial phenomenon. Acute mechanisms involve induction of negative intrathoracic pressure during the effort to breathe, which triggers recurrent episodes of hypoxia, hypercapnia, alterations in carbon dioxide and acid–base equilibrium, as well as surges in sympathetic nervous system activity. Chronic intermittent hypoxia (CIH) and negative thoracic pressure (NTP) induce atrial stretch, chronic structural remodeling, and elevated vagal tone, thereby heightening susceptibility to bradycardic and conduction arrhythmias. Intermediate pathways through which OSA may precipitate arrhythmia encompass heightened systemic inflammation, oxidative stress, a prothrombotic state, and vascular dysfunction. Long-term OSA is linked with atrial enlargement and fibrosis, ventricular hypertrophy, hypertension, and coronary artery disease. These factors predispose to cardiac arrhythmias through the following mechanisms: shortening of the atrial effective refractory period, abnormal automaticity, promotion of slowed and heterogeneous conduction, enhancement of reentrant arrhythmia persistence, and prolongation of the QT interval. In this paper, we aim to present the pathophysiological mechanisms underpinning the association between obstructive sleep apnea and cardiac arrhythmias. Understanding the precise pathophysiological pathways by which obstructive sleep apnea contributes to arrhythmogenesis will enable targeted preventive stratification of patients at risk for cardiovascular events and promote the development of innovative therapies to attenuate OSA-induced arrhythmogenicity.

## 1. Introduction

Obstructive sleep apnea (OSA) constitutes a chronic condition characterized by recurrent episodes of upper airway collapse during sleep. These episodes precipitate repeated nocturnal hypoxemia, sleep fragmentation, fluctuations in blood pressure, and heightened sympathetic nervous system activation.

The severity of obstructive sleep apnea is classified according to the apnea–hypopnea index (AHI), which represents the number of apneic and hypopneic events per hour of sleep: mild (AHI ≥ 5 to <15), moderate (AHI ≥ 15 to <30), and severe (AHI ≥ 30) [1,2,3,4].

Apneic events are defined as a >90% reduction in baseline airflow for a period of 10 s or more.

The 2014 American Academy of Sleep Medicine (AASM) define adult hypopnea as a greater than 30% decrease in nasal pressure from baseline amplitude for a period of 10 s or more. Additionally, AASM recommends that events be scored if they are accompanied by either an oxygen desaturation of 3% or more from the pre-event baseline or an associated arousal. Due to its elevated risk of morbidity and mortality, obstructive sleep apnea represents a major public health concern [1].

The prevalence of sleep apnea in the general population ranges from 9% to 38% and affects approximately 34% of middle-aged men and 17% of middle-aged women, with a substantial proportion of patients remaining undiagnosed [1]. This prevalence varies considerably depending on factors such as age, gender, body composition, and the specific definition of sleep apnea. Notably, at least 24% of individuals over the age of 65 suffer from obstructive sleep apnea, with its prevalence showing a marked increase over the past two decades [2,3]. Obstructive sleep apnea is frequently observed among patients with cardiac rhythm disorders and represents a strong independent risk factor for arrhythmias. Sex-related differences may additionally influence the cardiovascular and arrhythmogenic consequences of obstructive sleep apnea, particularly in postmenopausal women, and are discussed further in this review. OSA is particularly common among individuals with hypertension (exceeding 30%), heart failure (approximately 40%), and atrial fibrillation, with prevalence rates ranging from 40% to 50% in the latter [1,3,4].

Obstructive sleep apnea is associated with a wide range of arrhythmias, including both brady arrhythmias (sinus bradycardia, atrioventricular blocks) and tachyarrhythmias (atrial fibrillation, ventricular tachycardia). Consequently, patients with severe sleep apnea also face an increased risk of sudden death. Continuous positive airway pressure treatment (CPAP) has been shown to reduce arrhythmias in these patients.

Due to the high prevalence and comorbidity of obstructive sleep apnea in patients with cardiovascular diseases, a Scientific Statement from the American Heart Association (recently published in 2021) recommends screening (clinical, assessing medical history, using appropriate questionnaires and sleep apnea screening devices) for arrhythmias in patients with sleep apnea, as well as screening patients with arrhythmias for sleep apnea [1].

Certain studies indicate that the severity of sleep-disordered breathing (SDB) in individual patients is not stable over time, exhibiting substantial night-to-night variability that cannot be detected through a single overnight sleep assessment [2,3].

The central theme of this article is the complex relationship between OSA and the development of cardiac arrhythmias, with particular emphasis on the pathophysiological mechanisms that transform OSA from a simple respiratory disorder into an important proarrhythmogenic cardiovascular condition. The article aims to demonstrate that OSA directly contributes to the occurrence of both atrial arrhythmias, particularly atrial fibrillation, and ventricular arrhythmias and conduction disorders through a combination of acute and chronic mechanisms.

Several narrative reviews have previously addressed the association between OSA and cardiac arrhythmias, particularly atrial fibrillation and autonomic dysfunction. However, the present review expands beyond previous publications by providing a broader and updated integrative perspective on OSA-related arrhythmogenesis. In addition to classical mechanisms such as intermittent hypoxia, negative intrathoracic pressure swings, oxidative stress, and sympatho-vagal imbalance, this review summarizes emerging molecular and cellular pathways including inflammasome activation, mitochondrial dysfunction, endothelial senescence, epigenetic remodeling, microRNAs, exosome-mediated signaling, and epicardial adipose tissue inflammation.

Furthermore, unlike previous reviews predominantly focused on atrial fibrillation, the present manuscript discusses the heterogeneous spectrum of atrial, ventricular, and conduction arrhythmias associated with OSA, while also addressing individual arrhythmogenic susceptibility, obesity-independent mechanisms, fibrosis burden, sex differences, and translational biomarkers potentially useful for risk stratification. Finally, recent advances in therapeutic strategies and future precision medicine perspectives are also discussed in order to provide a more comprehensive and clinically oriented overview of OSA-related arrhythmogenic mechanisms.

## 2. Methods

For writing the narrative review, studies elucidating the interplay between cardiac arrhythmias and obstructive sleep apnea were identified through searches of bibliographic databases, including PubMed, MEDLINE, and Cochrane, from dates 1995 to 2025–1st of June.

All articles related to possible arrhythmias (but focusing especially on atrial ones), arrhythmogenic mechanisms, and their relationship with obstructive sleep apnea were considered and reviewed.

Included studies encompassed meta-analyses, randomized controlled trials, case–control studies, observational studies, and systematic reviews. The following keywords were used in this search, in various combinations: “OSA”, “obstructive sleep apnea”, “sleep-disordered breathing”, “arrhythmia”, “arrhythmogenic mechanisms”, “atrial fibrillation”,” premature atrial contraction”. We have conducted a rigorous review of the papers and removed duplicates.

Only English-language articles with available abstracts were considered.

## 3. Arrhythmic Risk in Obstructive Sleep Apnea

Susceptibility to arrhythmias in patients with OSA is primarily due to structural and electrical remodeling of the heart, promoted by excessive negative intrathoracic pressure and increased sympathetic activation. These changes create an arrhythmogenic substrate for triggers arising from apneas and hypopneas [5,6].

Key observational and cohort studies supporting the association between obstructive sleep apnea and arrhythmias are summarized in Table 1.

Obstructive sleep apnea frequently coexists with cardiovascular diseases (CVD) through complex bidirectional mechanisms [5]. More than three decades ago, Guilleminault et al. reported a detailed case series of 400 patients with diagnosed OSA who underwent 24 h Holter electrocardiography. The study revealed that 48% of these patients displayed arrhythmias and conduction anomalies during sleep, with prominent rhythm disturbances including non-sustained ventricular tachycardia (VT), sinus arrest, and second-degree atrioventricular conduction block [6].

Multiple studies indicate that obstructive sleep apnea is associated with both supraventricular and ventricular tachyarrhythmias, although the latter are more prevalent in the presence of concomitant cardiac comorbidities, such as ischemic heart disease or heart failure [7].

Atrial fibrillation (AF) represents the most common sustained arrhythmia, conferring substantial morbidity, elevated stroke risk, diminished quality of life, and increased mortality [8]. A high prevalence of sleep-disordered breathing has been observed in young patients with paroxysmal and persistent atrial fibrillation, although preserved left ventricular (LV) function was found (62% vs. 38% in those without AF) [9].

Backer et al. showed cyclical variation in the heart rate in most patients with sleep apnea. Episodes of heart block were identified in 20% of patients with severe sleep apnea [10]. The association between obstructive sleep apnea and arrhythmias is presented in Table 1. Although the included studies consistently demonstrated an association between obstructive sleep apnea and cardiac arrhythmias, several methodological limitations should be considered. Therefore, a critical appraisal of the included studies was performed and is summarized in Table 2.

**Table 1:** Key observational and cohort studies supporting the association between obstructive sleep apnea and arrhythmias.

References	Study Design	Sample Size	Objectives of the Study	Results
Becker et al. (1998) [10]	cross-sectional	239	To assess the prevalence of heart block in patients with OSA	Most patients exhibited cyclical variations in heart rate. Episodes of heart block were identified in 17 out of 97 patients with severe OSA
Gami et al. (2005) [11]	retrospective	112	To assess the rates of sudden death from cardiac causes among people with OSA	Patients with OSA exhibited a 2.57-fold higher relative risk of sudden cardiac death (SCD) during the midnight to 6 a.m. interval compared to the rest of the day. In patients with OSA, 46% of SCD occurred between midnight and 6 a.m. The risk of nocturnal sudden death demonstrated a direct correlation with OSA severity, as quantified by the apnea-hypopnea index (AHI).
Mehra et al. (2006) [12]	cross-sectional	566	To establish the prevalence of nocturnal cardiac arrhythmias in individuals with and without OSA	Individuals with severe sleep-disordered breathing demonstrated two- to fourfold higher odds of complex arrhythmias compared to those without sleep-disordered breathing.
Tanigawa et al. (2006) [13]	prospective	1312	To investigate the association between sleep-disordered breathing and the risk of cardiac arrhythmias in middle-aged adults	Moderate-to-severe SDB was independently associated with a significantly increased risk of complex ventricular arrhythmias and atrial fibrillation, particularly in patients with nocturnal hypoxemia.
Gami et al. (2007) [14]	retrospective	3542	To determine whether obesity and OSA serve as independent predictors of incident atrial fibrillation or atrial flutter	Obesity and the magnitude of nocturnal oxygen desaturation were independent risk factors for incident atrial fibrillation in individuals < 65 years of age.
Garrigue et al. (2007) [15]	cross-sectional	98	To assess the prevalence and consequences of OSA in pacemaker patients	In paced patients, there was an excessively high prevalence of undiagnosed OSA (59%).
Monahan et al. (2009) [16]	prospective	767	To evaluate the association between OSA and nocturnal cardiac arrhythmias in older adults	SDB was significantly associated with atrial fibrillation and complex ventricular ectopy, particularly in patients with severe nocturnal hypoxemia.
Gami et al. (2013) [17]	prospective	10,701	To assess the risk of sudden cardiac death associated with OSA	OSA was a risk factor for sudden cardiac death. SCD was best predicted by age > 60 years, apnea–hypopnea index > 20, mean nocturnal O_2_ sat < 93%, lowest nocturnal O_2_ sat < 78%.
Holmqvist et al. (2015) [18]	retrospective	10,132	To assess whether patients with OSA exhibit a higher likelihood of progressing to more persistent forms of AF or experiencing worse outcomes compared with patients without OSA	Patients with OSA exhibited higher rates of severe symptoms, major adverse cardiovascular outcomes, and atrial fibrillation progression, but similar mortality and increased hospitalization risks (HR, 1.12 [95% CI, 1.03–1.22]; *p* = 0.0078).
Cadby et al. (2015) [19]	retrospective	6841	To determine whether OSA constitutes an independent risk factor for AF, beyond obesity and other established risk factors	OSA diagnosis and severity was independently associated with incident atrial fibrillation AHI > 5/h: HR, 1.55 (95% CI, 1.21–2.00); log(AHI + 1): HR, 1.15 (95% CI, 1.06–1.26); and log(time with SaO_2_ < 90%) + 1: HR, 1.12 (95% CI, 1.06–1.19).
Selim et al. (2016) [20]	cross-sectional	697	To assess whether OSA is associated with cardiac arrhythmias in individuals with multiple cardiovascular comorbidities and severe sleep-disordered breathing	There was an independent association between OSA and nocturnal cardiac arrhythmias, with greater SDB severity linked to higher risk of any arrhythmia. Compared to individuals without SDB, patients with moderate–severe SDB exhibited nearly threefold unadjusted odds of any cardiac arrhythmia (2.94; CI 95%, 2.01–4.30; *p* < 0.0001), along with twofold odds of tachyarrhythmias (2.16; CI 95%,1.47–3.18; *p* = 0.0011), cardiovascular events (2.01; 1.36–2.96; *p* = 0.003), and implantable cardioverter–defibrillator therapies (2.50; 1.58–3.95; *p* = 0.001).
Reshetnik et al. (2019) [21]	prospective	202	To determine the prevalence of OSA during the subacute phase of acute myocardial infarction, and to assess the impact of SDB severity on the prevalence of nonsustained ventricular tachycardia	The prevalence of SDB was elevated among patients with uncomplicated AMI. Severe SDB (AHI > 23/h) was independently associated with an increased risk of nonsustained ventricular tachycardia (NSVT).

OSA = obstructive sleep apnea, SCD = sudden cardiac death; AHI = apnea–hypopnea index; SDB = sleep-disordered breathing; NSVT = nonsustained ventricular tachycardia, AMI = acute myocardial infarction.

**Table 2:** Critical appraisal of the included studies.

Study	Strengths	Limitations
Becker et al. (1998) [10]	One of the first studies evaluating the association between OSA and cardiac conduction abnormalities; objective sleep assessment	Small sample size; cross-sectional design limits causal interpretation
Gami et al. (2005) [11]	Clinically relevant evaluation of nocturnal sudden cardiac death; clear association with OSA severity	Retrospective design; relatively small cohort
Mehra et al. (2006) [12]	Included comparison groups with and without OSA; comprehensive arrhythmia assessment	Cross-sectional design; inability to establish temporal relationship
Tanigawa et al. (2006) [13]	Prospective design; large population-based cohort	Potential residual confounding factors
Gami et al. (2007) [14]	Large sample size; analysis of independent predictors of atrial fibrillation	Obesity and cardiovascular comorbidities may influence results
Garrigue et al. (2007) [15]	Focused on pacemaker patients, an underexplored population	Limited generalizability due to selected population
Monahan et al. (2009) [16]	Prospective evaluation in older adults; assessment of nocturnal hypoxemia	Study population limited to elderly individuals
Gami et al. (2013) [17]	Very large prospective cohort; strong statistical power	Observational design cannot confirm causality
Holmqvist et al. (2015) [18]	Large cohort with long-term cardiovascular outcomes	Retrospective nature; possible selection bias
Cadby et al. (2015) [19]	Adjusted for obesity and other confounders; robust statistical analysis	Potential residual confounding despite adjustments
Selim et al. (2016) [20]	Included patients with severe cardiovascular disease; detailed arrhythmia evaluation	Multiple comorbidities may confound associations
Reshetnik et al. (2019) [21]	Evaluated OSA in acute myocardial infarction patients; clinically important outcomes	Small sample size; limited external validity

Overall, most included studies were observational in nature, which limits the ability to establish causality between obstructive sleep apnea and cardiac arrhythmias. However, several large cohort studies demonstrated consistent associations between OSA severity, nocturnal hypoxemia, and adverse cardiovascular outcomes. Differences in study populations, diagnostic criteria, and arrhythmia assessment methods may contribute to heterogeneity among findings.

## 4. Mechanisms of Arrhythmogenesis in OSA

**Figure 1:**
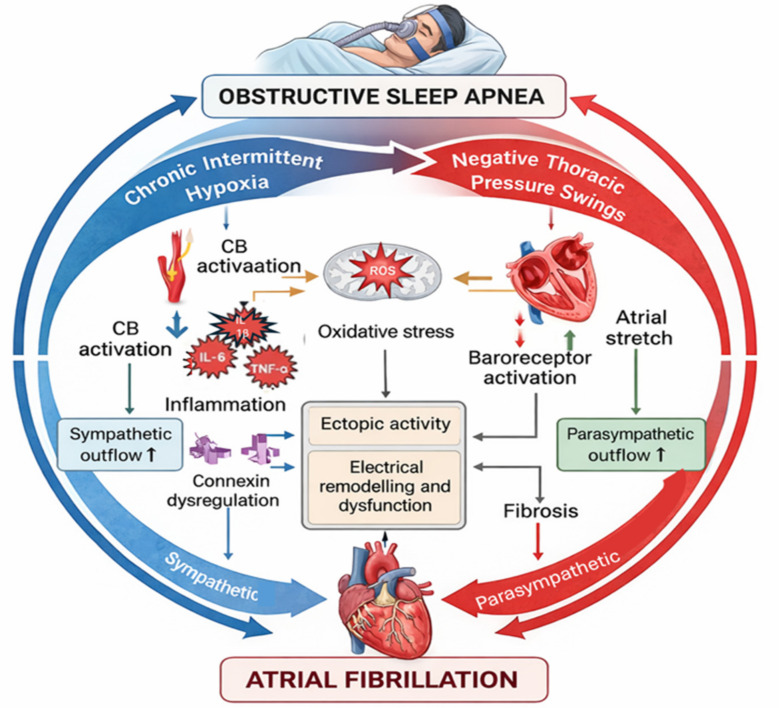
The mechanisms contributing to arrhythmia development in obstructive sleep apnea. Recurrent airway obstruction causes chronic intermittent hypoxia that facilitates atrial remodeling through inflammation-driven and oxidative stress-mediated processes. Enhanced peripheral chemoreceptor signaling increases sympathetic outflow to the heart, thereby facilitating ectopic activity and promoting long-term remodeling of cardiomyocyte ion channel expression. Negative intrathoracic pressure swings during apneic episodes induce additional structural remodeling and ectopic activity via atrial stretch, while baroreceptor activation acutely shortens the atrial effective refractory period (AERP), thereby favoring re-entry. Abbreviations: ROS: reactive oxygen species; CB: carotid body; HIF: hypoxia-inducible factor; IL: interleukin; TNF-α: tumor necrosis factor-α [22].

Several mechanisms involved in OSA-induced myocardial injury include systemic inflammation, neurohumoral activation, chronic atrial enlargement caused by repetitive intrathoracic pressure fluctuations, and associated comorbidities such as obesity and hypertension. It is believed that three major mechanisms initiate a cascade of intermediate processes leading to cardiac electrical abnormalities and an increased susceptibility to arrhythmias [22,23] (Figure 1). These mechanisms consist of negative intrathoracic pressure swings occurring during inspiratory efforts against an obstructed airway, chronic intermittent hypoxia accompanied by hypercapnia, and sympathetic nervous system activation triggered by recurrent arousals from sleep at the end of obstructive respiratory events.

In Table 3, a synthesis of the intricate mechanisms described in the aforementioned studies is presented.

### 4.1. Negative Thoracic Pressure Swings

Obstructive apnea, determined by upper airway collapse, leads to intrathoracic pressure swings followed by changes in the intracardiac transmural pressure gradient [22,23].

In patients with obstructive sleep apnea, negative tracheal pressures as low as −80 to −100 mbar have been documented [24,25]. A porcine model simulating OSA tracheal occlusion under an applied negative tracheal pressure of −80 mbar produced a right atrial pressure of −16 mbar and a negative intrathoracic pressure of −65 mbar [26]. These observations indicate heightened atrial transmural pressure gradients, leading to atrial distension. Analogous changes in intrathoracic pressure and atrial dimensions have been reported in humans during repetitive Müller maneuvering (attempted inspiration against the obstructed upper airways) [27].

These large oscillation pressures (up to –65 mmHg) lead to increased left ventricular afterload and compromise the thin-walled, compliant atria by causing acute distension [28]. Atrial distension leads to acute shortening of the atrial effective refractory period (AERP) via vagal activation [23,28,29,30]. In addition, these acute changes lead to increased central venous volume [31,32]. Abrupt application of marked negative intrathoracic pressure provokes a sudden reduction in left atrial volume and impairs left ventricular systolic performance [33,34]. Cyclical fluctuations in intrathoracic pressures induce acute distension of the thin-walled atria and pulmonary veins [35,36,37,38,39,40,41,42], potentially triggering stretch-mediated ion channel activation; concomitant variations in end-expiratory lung volumes and venous return may further contribute to arrhythmogenesis [43,44].

Shortened atrial refractoriness, coupled with reduced conduction velocity and increased heterogeneity in local conduction, enlarges the excitable gap, potentially heightening susceptibility to atrial fibrillation [45].

In healthy control subjects, simulated OSA via the Müller maneuver increased the frequency of premature beats and prolonged the corrected QT interval [46]. Progressive increases in atrial premature beat frequency coupled with prolongation of the corrected QT interval [47,48] have been implicated as precursors to AF [49,50] and ventricular arrhythmia/sudden cardiac death [51,52,53]. Upper airway occlusion impairs myocardial mechanics, leading to a characteristic reduction in left and right ventricular deformation during systolic cardiac contraction [53,54,55]. These ventricular disturbances may precipitate fluctuations in afterload, left ventricular hypertrophy, and heightened arrhythmogenic risk [56,57,58]. Increased transmural pressure gradients result in elevated atrial wall stress due to obstructive swings in intrathoracic pressure, heightened sympathetic activation triggered by arousals from sleep, and reactive oxygen species (ROS) induced by cyclical changes in hypoxemia with reoxygenation [59,60,61,62,63]. These factors have each been independently associated with enhanced CaMKII activity [64,65,66,67,68,69,70], which has previously been implicated in atrial arrhythmogenesis. Acute left atrial stretch, as occurs during apnea, is linked to Ca^2+^ overload, thereby triggering ectopic activity. Prolonged atrial stretch promotes structural and inflammatory remodeling, establishing a vulnerable substrate for re-entrant circuits [71,72].

### 4.2. Chronic Intermittent Hypoxia

Chronic intermittent hypoxia induces atrial and ventricular structural remodeling and promotes cardiac arrhythmias directly or indirectly through autonomic nervous system effects, local and systemic inflammation, and oxidative stress.

Intermittent cycles of deoxygenation–reoxygenation precipitate heightened production of reactive oxygen species, vascular inflammation, and elevated blood pressure, thereby promoting myocardial damage and cardiac remodeling [73,74]. The transition from hypercapnia to eucapnia during apneic events rapidly restores normal atrial refractoriness, whereas atrial conduction time remains prolonged and this temporal mismatch establishes an electrical milieu conducive to the initiation of atrial fibrillation [75,76]. Hypocapnia, specifically linked to OSA, can increase risk to develop arrhythmias by causing increased electrical instability [77,78]. Hypoxia can stimulate the sympathetic nervous system via reflex mechanisms. Prolonged exposure to hypoxia and hypercapnia can precipitate arrhythmias through pulmonary vascular remodeling, which culminates in right ventricular hypertension, right atrial dilatation, and potentially elevated transmural pressures on endocardial vessels. Hypoxemia arising from apneas and hypopneas is invariably accompanied by reoxygenation; these recurrent perturbations engender heightened inflammation and generation of oxygen-derived free radicals, culminating in tissue injury [55]. Moreover, hypoxia and oxygen-derived free radicals impair cardiac myocytes and disrupt myocyte ion exchange, thereby increasing the risk of cardiac dysfunction and facilitating arrhythmogenesis. Upregulation of systemic inflammation pathways may constitute a common final pathway that integrates autonomic dysfunction, hypoxia/hypercapnia and mechanical cardiac effects stemming from progressively negative intrathoracic pressures [79]. Hypercapnia has been demonstrated to alter the atrial refractory period; this pattern has been implicated in the increased vulnerability to atrial fibrillation observed in patients as they return to eucapnia [49,79].

**Figure 2:**
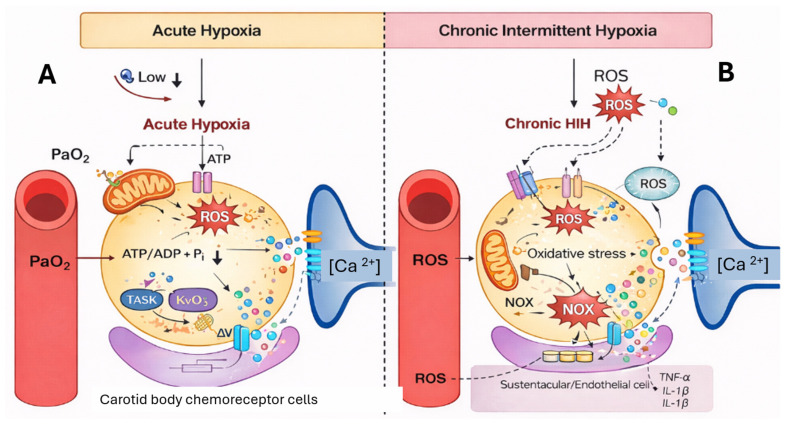
Schematic overview of the proposed ROS origins and their effects in acute oxygen sensing and under conditions of chronic intermittent hypoxia (CIH). (**A**): According to the prevailing hypothesis of oxygen sensing in carotid body chemoreceptor cells, acute hypoxia suppresses mitochondrial electron transport and/or oxidative phosphorylation, resulting in reduced ATP levels or alterations in the ATP/ADP + Pi ratio, along with increased ROS and NADH production. These changes inhibit background K^+^ (TASK) and oxygen-sensitive voltage-gated K^+^ (KvO_2_) channels, respectively, leading to membrane depolarization, Ca^2+^ influx, and subsequent release of excitatory neurotransmitters. (**B**): Chronic intermittent hypoxia (CIH) elevates ROS levels in carotid body chemosensory cells—and likely in adjacent endothelial and sustentacular cells—thereby promoting the expression of proinflammatory cytokines such as tumor necrosis factor-α (TNF-α), interleukin-1β (IL-1β), and interleukin-6 (IL-6). The resulting oxidative stress and proinflammatory environment increase intracellular Ca^2+^, leading to enhanced release of excitatory neurotransmitters and elevated chemosensory discharge. Adapted from [85].

In OSA, recurrent episodes of apnea lead to arterial oxygen desaturations that cyclically recur during the night. Episodes of intermittent hypoxia cause the release of ROS and sympathetic nervous system hyperreactivity [70,80,81,82,83,84] (Figure 2).

Under physiological conditions, hypoxemia stimulates the carotid body, triggering signals to the brainstem that induce both hyperventilation (to increase oxygen intake) and sympathetic activation (to raise blood pressure/flow) to restore adequate oxygen supply to tissues [70,80,81,83]. Upper airway obstruction prevents lung inflation, inhibiting the normal stretching of vagolytic fibers. This leads to increased parasympathetic tone and bradycardia, which, alongside sympathetic activation (the “diving reflex”), cause peripheral vasoconstriction to muscles and viscera, thereby rerouting oxygen to the brain and heart [62,85].

This reflex serves as a protective mechanism, but its chronic activation—as seen in obstructive sleep apnea—stresses the cardiovascular system, elevating risks for arrhythmias, hypertension, heart failure and stroke. The resultant hyperpnea upon arousal stretches peripheral afferent fibers in the lung, eliciting a vagolytic response (Herning–Breuer reflex) that, together with arousal-related increases in sympathetic tone, raises heart rate [70,80,82,83]. Research conducted on rodents’ exposure to intermittent hypoxia leads to an increased density of noradrenergic terminals in the trigeminal motor nucleus and the spinal trigeminal sensory nucleus, which can lead to altered transmission of cardiorespiratory and motor reflexes [86,87]. Intermittent hypoxia activates carotid bodies and the brainstem, boosting sympathetic nerve activity, which then directly stimulates adrenal chromaffin cells to release catecholamines causing systemic effects like increased heart rate and blood pressure to improve oxygen delivery [88,89].

Chronic intermittent hypoxia sensitizes this response, leading to heightened baseline sympathetic activity, hypertension, and increased catecholamine levels at the end of each apneic event [90]. In patients with obstructive sleep apnea, sympathetic tone is markedly increased along with a decrease in parasympathetic tone, yet surges in vagal tone are also observed This autonomic variability manifests characteristically as a bradycardia–tachycardia response, with bradycardia during apneic episodes and tachycardia upon their termination [48,91,92,93,94,95,96,97,98,99,100].

Ventricular premature beats are primarily linked to heightened sympathetic nervous system. Bradyarrhythmia, by contrast, arises from increased vagal tone triggered by apneic episodes. Parasympathetic predominance can provoke atrioventricular block and asystole, even in the absence of structural heart disease [6]. The central circadian clock modulates cardiac electrophysiology and arrhythmogenesis via the autonomic nervous system, while the intrinsic cardiac clock directly modifies ion channels (like HCN4, SCN5A), thereby altering the heart’s electrical properties; both pathways heighten arrhythmia risk by reshaping the underlying electrical substrate [101]. Sleep fragmentation and reduced sleep duration associated with OSA may represent a key factor, resulting in an enhanced state of systemic inflammation and oxidative stress—as evidenced by elevated myeloperoxidase and oxidized low-density lipoprotein levels, along with endothelial dysfunction and hypercoagulability [72,102]. Acute and subacute periods of sleep-disordered breathing impose repetitive cardiac mechanical stresses on the heart, resulting in atrial distention; elevated left ventricular pressure and transmural gradients; augmented venous return; heightened systemic inflammation and oxidative stress; and electrophysiological alterations (reduced atrial effective refractory period, QT prolongation, increased delayed afterdepolarizations and early afterdepolarizations [103,104].

Inflammatory biomarkers, such as adhesion molecules (Intercellular Adhesion Molecule-1 = ICAM-1, Vascular Cell Adhesion Molecule-1 = VCAM-1, selectins), C-reactive protein, interleukin-6 (IL-6), interleukin-8 (IL-8), fibrinogen, and tumor necrosis factor-alpha, have been associated with OSA [105,106,107,108,109,110]. A meta-analysis revealed elevated levels of C-reactive protein, tumor necrosis factor-α, IL-8, intercellular adhesion molecule, selectin, and vascular cellular adhesion molecule in individuals with OSA compared to control subjects [109,110]. Moreover, increasing soluble IL-6 receptor levels, considered to operate by more expansive trans-signaling pathways than IL-6, are associated with greater OSA severity, exhibiting a diurnal pattern independent of obesity [111,112]. Atrial fibrosis is a common response to CIH, and this is consistent with reports of upregulated transforming growth factor beta (TGF-β), connective tissue growth factor (CTGF), and α-smooth muscle actin [113,114,115,116,117,118,119,120,121,122,123]. These pro-fibrotic events are closely linked to the inflammatory response, with markers such as TNF-α, IL-6, and IL-1β extensively reported in both animal models and OSA patients [124,125]. The fibrotic substrate may be further aggravated by the remodeling of matrix metalo proteinase (MMP), specifically, the upregulation of MMP-9 and the downregulation of MMP-2 [126,127,128,129,130]. Thus, CIH drives an imbalance between collagen secretion and degradation. CIH may also contribute to atrial remodeling by promoting inflammatory myocardial apoptosis [130,131,132]. Repetitive cycles of hypoxia and reoxygenation also trigger the activation of the proinflammatory cascade, depletion of cellular adenosine 5′-triphosphate, and xanthine oxidase activation—all of which promote reactive oxygen species formation and nitric oxide bioavailability reduction [133,134,135].

In patients with OSA, leukocytes excessively generate ROS, which contribute to the development of an arrhythmogenic substrate [136]. OSA promotes the generation of deleterious ROS alongside the activation of proinflammatory cytokines and downregulation of anti-inflammatory mediators; these alterations induce endothelial dysfunction, predisposing individuals to cardiovascular disease and potentially augmented arrhythmia susceptibility. Due to their extended half-life, certain ROS can penetrate the endoplasmic reticulum and impair mitochondria—the cell’s principal energy-producing organelles—resulting in cardiomyocyte ultrastructural damage, inflammation, and myocardial degeneration. Collectively, these effects, compounded by electrolyte imbalances and perturbations in transmembrane voltage gradients, underline the arrhythmogenic remodeling of the myocardium [137,138,139,140,141,142].

Research confirms that rodents exposed to intermittent hypoxia experience significant heart injury, leading to changes like myocyte hypertrophy, altered cell length and apoptosis, with elevated serum cardiac troponin I (cTnI) levels serving as a biomarker for this damage. Long-term intermittent hypoxia causes cardiac remodeling, including hypertrophy and fibrosis [143,144,145]. In rats exposed to intermittent hypoxia, evidence of left ventricular dysfunction assessed by increased left ventricular end-diastolic pressure, impaired relaxation, and reduced contractility has been found; this ventricular dysfunction is linked to heightened oxidative stress, specifically increased lipid peroxides, alongside cellular injury such as myocyte hypertrophy and inflammation [146]. Such structural alterations of the myocardium may lead to micro-ischemia, promoting cardiac repolarization abnormalities and thus increased susceptibility to develop ventricular dysrhythmias [147,148]. Augmented oxidative stress heightens the risk of cardiac arrhythmia by disrupting mitochondrial function, leading to energy (adenosine triphosphate = ATP) depletion, unstable mitochondrial membrane potential, and imbalances in key ions (Ca^2+^, K^+^), metabolites (nicotinamide adenine dinucleotide, reduced form = NADH, adenosine diphosphate = ADP) and tricarboxylic acid cycle intermediates. These changes compromise mitochondrial respiration and energy production, fostering inhomogeneous excitability in heart tissue, and promoting re-entry arrhythmias and microvascular ischemia [149,150,151,152,153,154,155,156,157,158]. In addition, not only does the overproduction of ROS, induced by intermittent hypoxia, cause cellular damage (lipids, proteins, deoxyribonucleic acid = DNA), but ROS also act as signaling molecules. These ROS, generated by NADPH oxidases and mitochondria in the carotid bodies, trigger sympathetic responses and catecholamine release from the adrenal medulla [74,153]. In OSA patients, neurohumoral activation, particularly involving the circulating renin–angiotensin–aldosterone system concomitant with heightened oxidative stress, has been observed, potentially underlying atrial structural and electrical remodeling [159,160]. The renin–angiotensin–aldosterone system (RAAS) plays a significant role in promoting atrial structural remodeling across various pathophysiological conditions [45]. Hypoxemia directly activates chemoreceptors within the carotid body, eliciting augmented ventilation and sympathetic discharges [107,161,162,163]. Hypoxia additionally induces peripheral vasoconstriction, elevating both preload and afterload, and thereby causing increased cardiac workload. Hypoxia-induced oxidative stress uncouples endothelial nitric oxide synthase, promoting superoxide generation while reducing nitric oxide production [163]. Oxidative stress induces fibroblast-to-myofibroblast activation, promoting perivascular and interstitial fibrosis that slows conduction [164,165]. Obstructive sleep apnea is associated with increased epicardial adipose tissue (EAT) via hypoxia-induced inflammatory remodeling of adipose depots. This EAT releases inflammatory mediators (cytokines) and promotes atrial fibrosis, thereby establishing a vulnerable substrate for arrhythmias [166]. Intermittent hypoxia in OSA has also been associated with increased levels of prothrombotic markers, platelet activation, elevations of fibrinogen, increases in plasminogen activator inhibitor-1, platelet aggregation, and thrombus formation [167,168,169,170,171,172,173,174].

### 4.3. Emerging Molecular and Cellular Mechanisms

Beyond the classical mechanisms of OSA-related arrhythmogenesis, including intermittent hypoxia, negative intrathoracic pressure swings, oxidative stress, and autonomic imbalance, recent evidence suggests the involvement of complex molecular and cellular pathways that contribute to myocardial injury, fibrosis, electrical remodeling, and arrhythmia susceptibility. Chronic intermittent hypoxia (CIH), considered one of the principal pathophysiological hallmarks of OSA, initiates a cascade of inflammatory, metabolic, epigenetic, and mitochondrial alterations that may amplify the arrhythmogenic substrate.

These emerging mechanisms include inflammasome activation, mitochondrial dysfunction, endothelial senescence, epigenetic remodeling, exosome-mediated signaling, and epicardial adipose tissue inflammation.

#### 4.3.1. Inflammasome Activation

Recent studies indicate that chronic intermittent hypoxia activates innate immune pathways involved in cardiovascular remodeling and arrhythmogenesis. Among these, activation of the NOD-like receptor pyrin domain-containing protein 3 (NLRP3) inflammasome appears particularly important [175,176,177]. Recurrent hypoxia–reoxygenation cycles increase mitochondrial ROS production and activate nuclear factor kappa B (NF-κB), thereby promoting assembly of the NLRP3 inflammasome complex and subsequent caspase-1 activation [175,176].

Activated caspase-1 induces cleavage of pro-interleukin (IL)-1β and pro-IL-18 into their mature inflammatory forms, amplifying myocardial inflammation and oxidative injury [177]. Persistent inflammasome activation promotes fibroblast proliferation, extracellular matrix deposition, and collagen accumulation, contributing to atrial and ventricular fibrosis. Structural remodeling induced by inflammatory signaling may alter connexin expression, slow electrical conduction, and increase conduction heterogeneity, thereby facilitating re-entry circuits and AF maintenance [176].

Experimental evidence additionally suggests that inflammasome-mediated pyroptosis contributes to cardiomyocyte death and ventricular electrical instability [178]. Importantly, several studies indicate that inhibition of NLRP3 signaling attenuates atrial fibrosis and reduces AF susceptibility, supporting the hypothesis that inflammasome pathways may represent future therapeutic targets in OSA-related arrhythmogenesis [176].

#### 4.3.2. Mitochondrial Dysfunction

Mitochondrial dysfunction has emerged as another major contributor to OSA-induced cardiac electrical instability. Chronic intermittent hypoxia disrupts mitochondrial oxidative phosphorylation and promotes excessive ROS generation at the level of the mitochondrial electron transport chain [179]. Increased oxidative stress results in mitochondrial membrane depolarization, opening of mitochondrial permeability transition pores, depletion of ATP, impaired calcium homeostasis, and activation of proapoptotic signaling pathways [180].

These alterations profoundly affect cardiomyocyte electrophysiology. Mitochondrial oxidative injury modifies sodium, potassium, and calcium channel activity, thereby facilitating early afterdepolarizations, delayed afterdepolarizations, and triggered activity. Moreover, mitochondrial ROS contribute to activation of CaMKII, which has been strongly implicated in atrial and ventricular arrhythmogenesis [181]. CaMKII-mediated phosphorylation of ion channels enhances intracellular calcium overload, electrical instability, and ectopic activity.

In addition, mitochondrial dysfunction contributes to myocardial fibrosis, impaired ventricular relaxation, and heterogeneous electrical conduction, thereby creating a vulnerable substrate for both atrial and ventricular arrhythmias [182]. Experimental models of intermittent hypoxia have demonstrated myocardial hypertrophy, apoptosis, ventricular dysfunction, and fibrosis associated with mitochondrial oxidative injury [183]. These findings support the concept that mitochondria represent not only targets of oxidative injury but also active amplifiers of arrhythmogenic signaling in OSA.

#### 4.3.3. Endothelial Senescence

Endothelial dysfunction is increasingly recognized as a central mechanism linking OSA with cardiovascular disease. Intermittent hypoxia induces oxidative stress, inflammation, and reduced nitric oxide bioavailability, thereby impairing endothelial-dependent vasodilation and promoting vascular stiffness [184,185]. Beyond functional impairment, recent evidence suggests that OSA may accelerate endothelial senescence and vascular aging.

Endothelial senescence refers to irreversible endothelial cell-cycle arrest accompanied by acquisition of a proinflammatory senescence-associated secretory phenotype (SASP) [186]. Senescent endothelial cells release inflammatory cytokines, adhesion molecules, growth factors, and profibrotic mediators that contribute to vascular remodeling, hypertension, microvascular dysfunction, and myocardial fibrosis [187]. These alterations may indirectly facilitate arrhythmogenesis through atrial stretch, ischemia, autonomic dysregulation, and impaired myocardial perfusion.

Reduced nitric oxide bioavailability and endothelial oxidative injury may additionally impair coronary microcirculatory reserve and promote myocardial ischemia, thereby enhancing ventricular electrical instability. Furthermore, endothelial dysfunction contributes to prothrombotic states and platelet activation, which may further increase cardiovascular and thromboembolic risk in patients with AF and concomitant OSA [188].

#### 4.3.4. Epigenetic Remodeling

Epigenetic regulation has recently emerged as an important mediator of the long-term cardiovascular consequences of OSA. Chronic intermittent hypoxia may induce persistent alterations in gene expression through mechanisms including DNA methylation, histone modifications, chromatin remodeling, and regulation by non-coding ribonucleic acids (RNAs). These epigenetic modifications influence multiple pathways involved in inflammation, oxidative stress, fibrosis, autonomic regulation, and electrical remodeling.

Among non-coding RNAs, microRNAs (miRNAs) have attracted considerable attention as modulators of arrhythmogenic remodeling. miRNAs are small endogenous RNAs that regulate post-transcriptional gene expression and participate in numerous cardiovascular processes [189]. Intermittent hypoxia-induced dysregulation of miRNAs may promote atrial fibrosis, fibroblast proliferation, connexin dysfunction, calcium mishandling, and cardiomyocyte apoptosis, thereby contributing to atrial electrical instability and increased susceptibility to AF [190].

Several specific miRNAs, including miR-21, miR-29, miR-34a, and miR-155, have been associated with inflammatory and profibrotic signaling pathways in cardiovascular disease. For example, miR-21 is implicated in fibroblast activation and collagen deposition, whereas miR-29 regulates extracellular matrix remodeling. Altered expression of these miRNAs may therefore contribute to structural remodeling and conduction abnormalities observed in OSA patients [189].

Emerging evidence also suggests that circulating miRNAs may serve as biomarkers for cardiovascular risk stratification, OSA severity, and arrhythmia susceptibility [191]. Consequently, epigenetic pathways may represent promising therapeutic and diagnostic targets in future precision medicine approaches for OSA-related cardiovascular disease.

#### 4.3.5. Exosome-Mediated Signaling

Exosomes and extracellular vesicles are increasingly recognized as important mediators of intercellular communication in cardiovascular disease. Intermittent hypoxia associated with OSA alters exosomal cargo composition, including proteins, inflammatory mediators, lipids, messenger RNAs, and miRNAs [192]. These extracellular vesicles facilitate communication between endothelial cells, immune cells, adipocytes, fibroblasts, and cardiomyocytes, thereby propagating inflammatory and oxidative signaling pathways.

Experimental evidence suggests that exosomes generated during intermittent hypoxia promote endothelial dysfunction, oxidative stress, vascular inflammation, and myocardial remodeling [193]. Exosome-mediated transfer of proinflammatory and profibrotic miRNAs may additionally contribute to atrial fibrosis, connexin remodeling, and electrical instability [194]. In parallel, extracellular vesicles may amplify autonomic dysfunction and inflammatory signaling by modulating immune-cell activation and endothelial responses.

These observations suggest that exosome-mediated signaling may contribute to the systemic cardiovascular consequences of OSA and may represent a potential biomarker of disease severity and cardiovascular risk. Future therapeutic approaches targeting extracellular vesicle signaling pathways may offer novel opportunities for reducing OSA-related arrhythmogenic burden.

#### 4.3.6. Epicardial Adipose Tissue Inflammation

Recent evidence highlights the role of epicardial adipose tissue as an active inflammatory and arrhythmogenic organ in OSA. Chronic intermittent hypoxia promotes inflammatory remodeling of epicardial and pericardial adipose depots, increasing the release of adipokines, cytokines, reactive oxygen species, and profibrotic mediators.

Due to its close anatomical proximity to the atrial myocardium and the absence of fascial separation, inflamed epicardial adipose tissue exerts direct paracrine effects on adjacent atrial tissue. These effects include atrial fibrosis, oxidative stress, autonomic ganglion remodeling, slowed electrical conduction, and heterogeneous impulse propagation, thereby facilitating AF initiation and maintenance [195].

Inflammatory adipokines released from epicardial fat may additionally promote endothelial dysfunction, insulin resistance, and myocardial fibrosis, thereby amplifying cardiovascular remodeling in OSA patients. Increased epicardial adipose tissue volume has been independently associated with AF burden, AF recurrence after catheter ablation, and adverse cardiovascular outcomes [194].

Emerging data suggest that atrial adipose inflammation may represent an important mechanistic bridge linking obesity, metabolic dysfunction, OSA, and atrial arrhythmogenesis. Consequently, modulation of epicardial adipose tissue inflammation may become a future therapeutic target in patients with OSA-associated AF [193].

### 4.4. Sympatho-Vagal Imbalance

Sympathetic activation determined by recurrent arousals, intermittent hypoxia, oxidative stress, and intrathoracic pressure changes trigger constant sympathetic nervous system activity, leading to harmful electrical and structural changes in the heart, making it prone to dangerous arrhythmias like fibrillation.

In OSA, cardiac autonomic balance varies across the phases of acute apneic episodes. During the apneic phase, substantial bradycardia occurs, signifying parasympathetic dominance.

Following the apneic period, a rebound tachycardia ensues, signifying heightened cardiac sympathetic activity. Then, the heart rate gradually returns to baseline [196].

These autonomic fluctuations are likely mediated by arterial baroreceptors, peripheral chemoreceptors, central respiratory centers, and pulmonary afferents.

Pulse/heart rate variability (PRV/HRV) provides a valuable clinical assessment of autonomic balance. In OSA patients’ sleep, HRV metrics indicate a shift toward sympathetic predominance, evidenced by an elevated low frequency/high frequency (LF/HF) ratio [148,197]. Mechanistically, obstructive respiratory events may cause structural remodeling and myocardial damage through repetitive mechanical atrial distension and atrial wall stretch, as well as frequent episodes of oxyhemoglobin desaturation–resaturation. Long-term OSA has been associated with significant atrial remodeling, characterized by “atrial enlargement, local conduction disturbances and longer sinus node recovery” [196], atrial electromechanical delay, and left atrial dysfunction [197].

**Figure 3:**
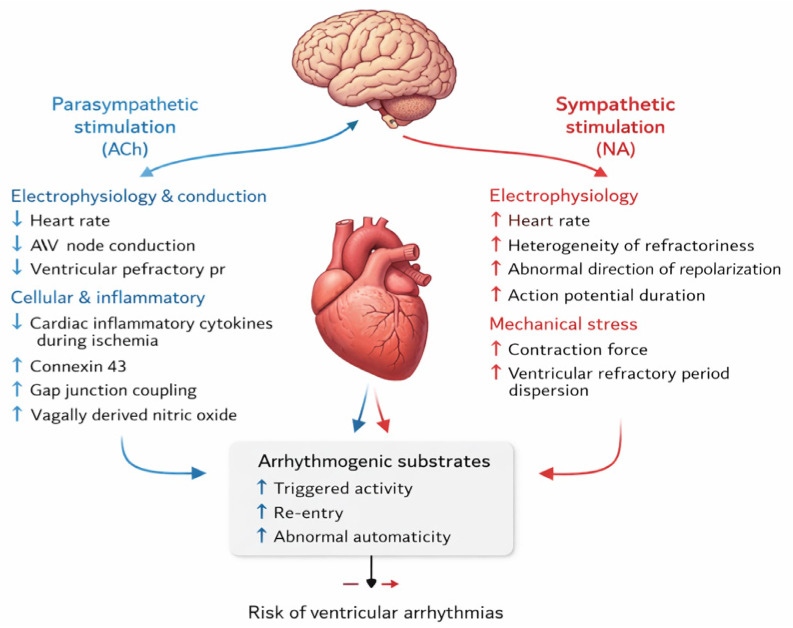
Sympathetic and parasympathetic nervous system-mediated cardiac arrhythmogenesis. Blue = parasympathetic nervous system. Red = sympathetic nervous system. Black = Links between the brain and the sympathetic and parasympathetic nervous systems. Up arrows = increase. Down arrows = decrease. Red line and + = provoke these events. Blue line and − = inhibit these events. Adapted from [199].

Acute hyperactivation of the sympathetic nervous system, from stress or conditions like sleep apnea, provoked coronary vasoconstriction, leading to micro-ischemia, which creates electrical instability, increasing dispersion and duration of myocardial repolarization, thereby raising the risk for arrhythmias and sudden cardiac death [198,199] (Figure 3). In OSA, the autonomic nervous system—via Ca/calmodulin-dependent protein kinase II (CaMKII)—can trigger arrhythmias by altering sodium channels. Activated by calcium signals, CaMKII phosphorylates these sodium channels, augmenting late sodium current, and thereby provoking delayed after depolarizations (DADs) and abnormal heartbeats. This pathway links autonomic nervous system activity to cellular clock dysfunction and circadian rhythms in the heart [200,201,202,203,204]. Hypoxia, acidosis, and ventricular hypertrophy are precipitating factors for early after-depolarizations. Delayed afterdepolarizations resulting in triggered activity often occur in response to increased catecholamine levels, which are also inherent to OSA [127,205].

Prolonged apneic episodes and hypoxia elicit intricate autonomic alterations marked by heightened parasympathetic tone, particularly during rapid eye movement sleep [206,207].

In OSA, temporary cessations in breathing cause hypoxia, which stimulates carotid body chemoreceptors, sending signals via the vagus nerve to slow the heart, resulting in bradycardia. How strongly this happens varies between people due to their chemosensitivity, hypoxia severity, and how their sinoatrial node is affected by hypoxia [96,208].

## 5. Why Do Only Some OSA Patients Develop Arrhythmias?

Although OSA is strongly associated with atrial and ventricular arrhythmias, not all patients with OSA develop clinically significant electrical disturbances. This observation suggests that OSA alone may not be sufficient to induce arrhythmogenesis, but rather acts as a trigger superimposed on an individual arrhythmogenic substrate shaped by genetic, structural, autonomic, inflammatory, metabolic, and sex-related factors [209,210].

Genetic predisposition may partially explain the heterogeneous arrhythmic response observed among patients with OSA. Variability in genes regulating ion-channel function, oxidative stress responses, inflammatory signaling, autonomic regulation, and myocardial fibrosis may influence susceptibility to AF and ventricular arrhythmias [211,212]. Polymorphisms involving sodium, potassium, and calcium channel genes may alter cardiac electrophysiological stability and increase vulnerability to triggered activity and re-entry mechanisms under hypoxic stress conditions. In addition, genetic variations affecting TGF-β, RAAS signaling, and inflammatory pathways may amplify myocardial fibrosis and structural remodeling induced by intermittent hypoxia [212]. Emerging evidence also suggests that inherited differences in oxidative stress handling and mitochondrial resilience may influence the severity of myocardial injury associated with OSA.

Considerable heterogeneity exists in autonomic nervous system responses among patients with OSA. Some individuals exhibit pronounced sympathetic activation and elevated chemoreflex sensitivity, whereas others demonstrate predominant vagal responses during apneic episodes [213,214]. Enhanced sympathetic activation may increase arrhythmic susceptibility through catecholamine-mediated triggered activity, calcium overload, QT prolongation, and ventricular electrical instability. Conversely, exaggerated vagal responses may facilitate atrial refractoriness shortening and bradyarrhythmias, including sinus pauses and atrioventricular conduction block.

Differences in autonomic phenotype may therefore explain why some patients predominantly develop atrial fibrillation, whereas others present with bradyarrhythmias or ventricular arrhythmias. Variability in carotid body sensitivity, baroreflex function, autonomic adaptability, and circadian autonomic regulation likely contributes to these divergent electrophysiological responses [214].

The severity of pre-existing atrial remodeling appears to play a critical role in determining arrhythmic susceptibility in OSA patients. Structural abnormalities including atrial enlargement, fibrosis, conduction slowing, connexin remodeling, altered calcium handling, and electromechanical dysfunction create a vulnerable substrate for AF initiation and maintenance [215,216].

Importantly, not all OSA patients exhibit the same degree of myocardial remodeling. Individuals with greater atrial fibrosis burden may be particularly susceptible to intermittent hypoxia-induced electrical instability [217]. Advanced fibrosis contributes to conduction heterogeneity, slowed impulse propagation, impaired cell-to-cell electrical coupling, and re-entry circuit formation, thereby facilitating persistent arrhythmias.

Moreover, coexisting cardiovascular conditions such as hypertension, heart failure, obesity, metabolic syndrome, and coronary artery disease may synergistically amplify structural remodeling and increase arrhythmogenic vulnerability [210,216]. The interaction between OSA-induced hypoxia and an already diseased atrial substrate likely explains the marked variability in arrhythmia burden observed among patients.

Although obesity is strongly associated with OSA and atrial fibrillation, increasing evidence suggests that OSA may promote arrhythmogenesis independently of obesity [218,219]. Several studies have demonstrated associations between intermittent hypoxia, autonomic dysfunction, oxidative stress, endothelial dysfunction, and atrial remodeling even after adjustment for body mass index and metabolic factors.

Non-obese patients with OSA may still develop significant arrhythmogenic remodeling through mechanisms involving sympathetic activation, systemic inflammation, oxidative stress, endothelial injury, and negative intrathoracic pressure swings. In these individuals, repetitive nocturnal hypoxemia may directly promote myocardial fibrosis, calcium-handling abnormalities, and electrical instability independently of adiposity [219].

These observations suggest that OSA itself represents an independent proarrhythmogenic condition rather than merely a marker of obesity-related cardiovascular risk.

Estrogen exerts several protective cardiovascular effects, including enhancement of endothelial nitric oxide production, attenuation of sympathetic nervous system activation, reduction in oxidative stress, and modulation of inflammatory signaling pathways. In premenopausal women, these mechanisms may partially protect against atrial remodeling, myocardial fibrosis, endothelial dysfunction, and electrical instability. However, after menopause, estrogen deficiency may promote endothelial dysfunction, increased oxidative stress, autonomic imbalance, vascular stiffness, and profibrotic remodeling, thereby increasing susceptibility to atrial fibrillation and other cardiac arrhythmias [220].

In addition, sex-related differences in fat distribution, inflammatory responses, atrial structure, autonomic regulation, and electrophysiological properties may contribute to heterogeneous arrhythmogenic phenotypes among OSA patients [221]. Postmenopausal women frequently exhibit underrecognized OSA manifestations and may experience delayed diagnosis despite substantial cardiovascular risk.

These findings suggest that biological sex and hormonal status may substantially modulate the cardiovascular and arrhythmogenic consequences of OSA and should be considered in future individualized risk stratification and therapeutic approaches.

Overall, current evidence suggests that arrhythmia development in OSA depends on the interaction between intermittent hypoxia-related triggers and the presence of a susceptible arrhythmogenic substrate. Genetic predisposition, autonomic phenotype, fibrosis burden, obesity-independent mechanisms, metabolic dysfunction, and sex-related factors likely interact to determine individual arrhythmic vulnerability [222].

Consequently, future approaches may require personalized phenotyping strategies integrating clinical, imaging, electrophysiological, inflammatory, and molecular biomarkers in order to identify OSA patients at highest arrhythmic risk and optimize targeted therapeutic interventions.

## 6. Translational Biomarkers of Arrhythmic Risk in OSA

Given the heterogeneous cardiovascular manifestations of OSA, increasing attention has been directed toward the identification of translational biomarkers capable of improving arrhythmic risk stratification. Emerging biomarkers may help identify OSA patients at higher risk of atrial and ventricular arrhythmias, facilitate earlier therapeutic interventions, and potentially guide individualized management strategies. Current evidence suggests that inflammatory, oxidative, autonomic, and structural remodeling biomarkers may reflect the complex arrhythmogenic substrate associated with OSA.

Systemic inflammation and myocardial fibrosis represent central mechanisms linking OSA to arrhythmogenesis. Several circulating inflammatory biomarkers have therefore been investigated as potential indicators of cardiovascular and arrhythmic risk.

IL-6, a key proinflammatory cytokine elevated in OSA, has been associated with disease severity, endothelial dysfunction, atrial remodeling, and atrial fibrillation susceptibility [223,224]. Increased IL-6 signaling may promote fibroblast activation, extracellular matrix remodeling, oxidative stress, and autonomic dysregulation.

Galectin-3, a profibrotic lectin involved in fibroblast proliferation and collagen deposition, has emerged as another promising biomarker. Elevated galectin-3 levels have been associated with atrial fibrosis, atrial remodeling, heart failure progression, and recurrence of atrial fibrillation after catheter ablation [225]. Given the importance of fibrosis in OSA-related arrhythmogenesis, galectin-3 may reflect the extent of structural atrial remodeling induced by chronic intermittent hypoxia.

Similarly, soluble suppression of tumorigenicity-2 (sST2), a marker of myocardial stress, fibrosis, and inflammation, has demonstrated associations with adverse cardiovascular remodeling and arrhythmic risk [226]. Increased sST2 concentrations may indicate ongoing myocardial injury and profibrotic activation in OSA patients with advanced cardiovascular involvement.

The receptor for advanced glycation end products (RAGE) pathway has attracted growing attention in OSA-related cardiovascular disease. Soluble RAGE (sRAGE), considered a decoy receptor with anti-inflammatory properties, may reflect oxidative stress burden and endothelial dysfunction. Reduced circulating sRAGE levels have been associated with increased inflammation, vascular injury, and cardiovascular risk in patients with OSA.

Given the central role of oxidative stress and endothelial dysfunction in atrial remodeling and arrhythmogenesis, sRAGE may represent a promising biomarker for identifying patients with enhanced cardiovascular vulnerability [227].

Heart rate variability analysis represents a noninvasive tool for assessing autonomic nervous system dysfunction in OSA. HRV metrics provide insight into sympatho-vagal balance and may identify patients with increased arrhythmogenic susceptibility [228].

Patients with OSA frequently exhibit reduced parasympathetic activity and enhanced sympathetic predominance, reflected by increased low frequency/high frequency (LF/HF) ratios and impaired HRV parameters [229]. Reduced HRV has been associated with increased risk of atrial fibrillation, ventricular arrhythmias, sudden cardiac death, and adverse cardiovascular outcomes.

Moreover, HRV abnormalities may correlate with intermittent hypoxia severity, nocturnal desaturation burden, and sleep fragmentation, thereby representing a dynamic marker of autonomic remodeling and cardiovascular stress in OSA [230].

Advanced cardiac imaging techniques are increasingly used to identify subclinical atrial dysfunction in OSA patients. Speckle-tracking echocardiography-derived atrial strain imaging has emerged as a sensitive marker of atrial mechanical dysfunction and fibrosis [231].

Reduced left atrial strain has been associated with atrial enlargement, impaired atrial compliance, fibrosis burden, and increased atrial fibrillation risk [232]. In OSA, recurrent negative intrathoracic pressure swings, intermittent hypoxia, and autonomic dysfunction may progressively impair atrial mechanical function even before overt structural remodeling becomes apparent.

Consequently, atrial strain imaging may represent a valuable noninvasive biomarker for early identification of patients at increased arrhythmic risk and for monitoring the effects of therapeutic interventions such as continuous positive airway pressure (CPAP) therapy [36].

Although several translational biomarkers show promising associations with arrhythmogenic risk in OSA, their clinical utility remains incompletely established. Future research should focus on integrating circulating biomarkers, autonomic markers, imaging parameters, electrophysiological characteristics, and molecular profiling into multimodal risk stratification models.

Such approaches may improve identification of OSA patients at highest risk of atrial and ventricular arrhythmias and facilitate personalized preventive and therapeutic strategies.

## 7. Therapies Targeting Arrhythmic Burden in Obstructive Sleep Apnea

Given the multifactorial and heterogeneous mechanisms underlying obstructive sleep apnea (OSA)-related arrhythmogenesis, several therapeutic approaches have been proposed to reduce arrhythmic burden. Nevertheless, current evidence supporting these strategies remains relatively limited, as only a small number of studies have systematically evaluated their efficacy [233,234]. Therapeutic management generally aims not only to alleviate upper airway obstruction, but also to counteract the autonomic, inflammatory, hypoxic, and structural cardiac alterations associated with OSA.

Lifestyle modification represents a cornerstone in the management of OSA progression. Weight reduction achieved through dietary interventions, medical therapy, or bariatric surgery has been shown to improve OSA severity and indirectly reduce cardiovascular risk. However, these approaches may be associated with important limitations and adverse effects, including anesthetic complications, gastroesophageal reflux, chronic nausea and vomiting, or even failure to achieve significant weight loss.

Among available therapies, continuous positive airway pressure (CPAP) remains the gold standard treatment for OSA despite persistent challenges related to long-term patient adherence. CPAP appears to substantially improve cardiac rhythm disturbances through multiple mechanisms, including reduction in nocturnal hypoxemia, attenuation of autonomic dysfunction and oxidative stress, reversal of atrial remodeling, and mitigation of negative intrathoracic pressure swings [234,235]. Furthermore, although many published studies are limited by methodological constraints and relatively small sample sizes, current evidence suggests that CPAP therapy may reduce atrial fibrillation recurrence and improve the success rates of electrical cardioversion and catheter ablation in patients with atrial fibrillation [236]. CPAP has also been associated with a reduction in nocturnal ventricular arrhythmias, as well as a marked decrease or complete elimination of sinus bradycardia, sinus pauses, and atrioventricular block episodes. Collectively, these benefits suggest that adherent CPAP users experience lower mortality rates and fewer cardiovascular events compared with untreated or non-adherent patients. Nevertheless, treatment efficacy is strongly dependent on compliance, which remains a major limitation due to pressure intolerance, mask discomfort, claustrophobia, dryness of the mouth or nose, and skin irritation [1].

Alternative noninvasive therapeutic options include oral appliances, particularly mandibular advancement devices, which generally demonstrate superior patient compliance compared with CPAP, auto-titrating positive airway pressure (APAP), or bilevel positive airway pressure (BiPAP). However, their use may be limited by adverse effects such as myofascial discomfort and excessive salivation [1]. In parallel, antiarrhythmic drug therapy has also been explored, although the incompletely understood pathophysiological mechanisms of OSA, together with disease severity, may partly explain the reduced efficacy of agents such as flecainide or dronedarone, as well as the lower success rates of cardioversion and catheter ablation in patients with concomitant atrial fibrillation [237,238].

Increasing attention has also been directed toward therapeutic strategies targeting autonomic imbalance, which is considered a major contributor to OSA-related arrhythmogenesis [235]. Several modulation approaches have been proposed, including beta-blockers, which may reduce chronic OSA-induced atrial sympathetic hyperinnervation, adipokine release, cardiac apoptosis, fibrosis, and metabolic remodeling [188,233,239,240,241]. Additional strategies involve antidepressants that modulate serotonergic pathways [234], low-level vagus nerve stimulation aimed at attenuating atrial electrophysiological alterations, and low-level baroreflex stimulation or carotid body ablation, both of which blunt the ventilatory response to hypoxia and contribute to restoration of autonomic and baroreflex cardiac function [118]. Renal denervation has also emerged as a potential therapeutic option by reducing sympathetic outflow and attenuating OSA-induced autonomic, structural, and electrical atrial remodeling [242]. Similarly, ganglionated plexi ablation may improve autonomic imbalance and atrial electrophysiological abnormalities associated with OSA [237,238,243].

Finally, surgical interventions such as uvulopalatopharyngoplasty are generally reserved for patients in whom less invasive therapeutic strategies have failed, reflecting the need for individualized and stepwise management approaches in this complex patient population.

Recent advances in the understanding of OSA-related arrhythmogenic mechanisms have generated increasing interest in targeted therapeutic strategies extending beyond conventional CPAP therapy. Although CPAP remains the cornerstone of OSA management, emerging evidence suggests that additional approaches targeting autonomic imbalance, inflammation, oxidative stress, fibrosis, and metabolic dysfunction may further reduce arrhythmic burden.

Growing attention has been directed toward therapies modulating autonomic nervous system activity. Renal sympathetic denervation has demonstrated potential benefits in reducing sympathetic hyperactivity, blood pressure, atrial remodeling, and atrial fibrillation susceptibility in selected patients with OSA and resistant hypertension [210]. Similarly, low-level vagal nerve stimulation and ganglionated plexi modulation have emerged as experimental strategies capable of attenuating autonomic dysregulation and electrical remodeling associated with atrial fibrillation [244].

Anti-inflammatory and antifibrotic therapies are also increasingly investigated. Experimental studies suggest that inhibition of NLRP3 inflammasome signaling, oxidative stress pathways, and profibrotic mediators such as TGF-β may attenuate atrial fibrosis and electrical instability induced by intermittent hypoxia [176]. In addition, therapies targeting mitochondrial dysfunction and ROS production may potentially reduce cardiomyocyte injury and arrhythmogenic remodeling.

Weight reduction strategies, including glucagon-like peptide-1 receptor agonists (GLP-1RAs) and bariatric interventions, have recently gained interest due to their favorable effects on obesity, systemic inflammation, metabolic dysfunction, and OSA severity [245]. These interventions may indirectly reduce atrial remodeling and arrhythmic susceptibility. Furthermore, increasing evidence supports the role of integrated risk-factor management, including hypertension control, weight optimization, diabetes treatment, and lifestyle modification, in improving atrial fibrillation outcomes in OSA patients.

Advances in catheter ablation techniques and personalized electrophysiological approaches may additionally improve rhythm-control strategies in OSA-associated atrial fibrillation. However, OSA remains associated with higher AF recurrence rates after ablation, emphasizing the importance of concomitant treatment of sleep-disordered breathing [246].

## 8. Future Directions

Despite substantial progress in understanding OSA-related arrhythmogenesis, several important gaps remain incompletely elucidated. Future research should focus on better characterization of the molecular, inflammatory, autonomic, and electrophysiological mechanisms linking intermittent hypoxia with cardiac electrical remodeling.

Particular attention should be directed toward identifying patient-specific arrhythmogenic phenotypes through integration of genetic, molecular, imaging, autonomic, and biomarker profiling. Such precision medicine approaches may improve prediction of arrhythmic risk and facilitate individualized therapeutic strategies.

Additional studies are also needed to clarify the role of emerging molecular pathways, including inflammasome activation, mitochondrial dysfunction, epigenetic remodeling, extracellular vesicle signaling, and epicardial adipose tissue inflammation in OSA-related cardiovascular disease. The development of novel biomarkers capable of identifying subclinical atrial remodeling before overt arrhythmia manifestation may significantly improve early prevention strategies.

Future randomized clinical trials should further evaluate whether aggressive treatment of OSA and associated cardiometabolic abnormalities can effectively reduce atrial and ventricular arrhythmia burden, sudden cardiac death risk, and long-term cardiovascular outcomes. Moreover, the impact of novel pharmacological agents targeting oxidative stress, fibrosis, autonomic dysfunction, and inflammatory pathways warrants further investigation.

Finally, advances in artificial intelligence, wearable technologies, remote monitoring systems, and digital health platforms may facilitate continuous assessment of sleep-disordered breathing, autonomic dysfunction, and arrhythmia burden in real-world settings. These innovations may contribute to earlier diagnosis, dynamic risk stratification, and more personalized long-term management of patients with OSA-associated arrhythmogenic disease.

## 9. Conclusions

Obstructive sleep apnea is a major public health problem associated with increased cardiovascular morbidity and mortality. Considering the contemporary pertinence of this research domain and its substantial implications for clinical practice and public health, the dissemination of novel empirical data assumes critical importance.

This narrative review delineates the pathophysiological mechanisms underlying the association between obstructive sleep apnea and arrhythmic risk, including acute factors such as negative intrathoracic pressure swings, chronic intermittent hypoxia/hypercapnia, and sympathetic nervous system activation. OSA-induced inflammation, oxidative stress, oxygen-derived free radical production, and the modulation of autonomic nervous system activity substantially contribute to myocardial cellular injury and fibrosis, thereby fostering cardiac electrical instability and arrhythmia susceptibility.

In the therapeutic management of arrhythmias associated with OSA, implementing a bidirectional screening strategy (according to the American Heart Association current medical guidelines regarding Obstructive Sleep Apnea and Cardiovascular Disease [1]) is essential, involving OSA screening in patients with arrhythmia, but also arrhythmia screening in those with OSA.

As previously stated, the therapeutic impact on the occurrence of arrhythmias and on treatment response is still under evaluation, with the polymorphism of the underlying mechanisms leading to a multidirectional relationship between OSA, cardiac remodeling, and treatment modalities and response. Future research should prioritize clarifying the mechanistic links between sleep disorders, arrhythmias, and broader cardiovascular health to optimize patient management and therapeutic efficacy.

In conclusion, OSA extends well beyond a mere respiratory condition, with its cardiovascular implications firmly substantiated. Consequently, treatment strategies must be tailored, and thorough prognostic evaluations conducted by multidisciplinary pulmonary–cardiac teams are essential.

## Figures and Tables

**Table 3:** Synthesis of the intricate mechanisms involved in obstructive sleep apnea.

References	Study Design	Possible Proposed Mechanisms
Becker et al. (1998) [10]	cross-sectional	Chronic intermittent hypoxia and hypercapnia; sympathetic activation
Gami et al. (2005) [11]	retrospective	Hypoxemia; increased sympathetic activity; abnormalities in cardiac autonomic and electrophysiological factors
Gami et al. (2007) [14]	retrospective	Repetitive and prolonged hypoxemia; decrease in nocturnal oxygen saturation, exaggerated intrathoracic pressure oscillations with increased cardiac wall stress, sympathetic and parasympathetic imbalances, marked autonomic imbalance, diastolic dysfunction, rapid swings in cardiac transmural pressures
Garrigue et al. (2007) [15]	cross-sectional	Sleep apnea-induced hypoxemia, increased vagal tone
Mehra et al. (2006) [12]	cross-sectional	Alterations in sympathetic and parasympathetic nervous system activity, autonomic nervous system dysregulation, hypoxemia, acidosis, abnormal automaticity, triggered automaticity, and reentry mechanisms
Gami et al. (2013) [17]	prospective	Nocturnal oxygen desaturation—systemic hypoxemia associated with hypercapnia, increases vascular sympathetic nerve activity and serum catecholamines, cardiac autonomic dysfunction
Holmqvist et al. (2015) [18]	retrospective	The article did not discuss the involved mechanisms
Selim et al. (2016) [20]	cross-sectional	Repetitive episodes, sympathetic hyperactivity, significant intrathoracic pressure swings responsible for increased atrial and ventricular cardiac wall stress
Reshetnik et al. (2019) [21]	prospective	Impaired autonomic function, and sympathetic hyperactivity, hypoxia

## Data Availability

No new data were created or analyzed in this study. Data sharing is not applicable to this article.

## Abbreviations

AASM	American Academy of Sleep Medicine
AERP	atrial effective refractory period
AF	atrial fibrillation
AHI	apnea–hypopnea index
AMI	acute myocardial infarction
ANS	autonomic nervous system
ADP	adenosine diphosphate
APAP	auto titrating positive airway pressure
ATP	adenosine triphosphate
BiPAP	bilevel positive airway pressure
CaMKII	Ca/calmodulin-dependent protein kinase II
CB	carotid body
CIH	chronic intermittent hypoxia
CPAP	continuous positive airway pressure
CTGF	connective tissue growth factor
cTnI	cardiac troponin I
CVD	cardiovascular diseases
DADs	delayed afterdepolarizations
DNA	deoxyribonucleic acid
EADs	early afterdepolarizations
EAT	epicardial adipose tissue
GLP-1Ras	glucagon-like peptide-1 receptor agonists
HF	high frequency
HIF	hypoxia-inducible factor
HR	hazard ratio
HRV	heart rate variability
ICAM-1	intercellular adhesion molecule-1
IL/IL-1β/IL6/IL-8	interleukin/interleukin-1β/interleukin-6/interleukin-8
KvO_2_	oxygen-sensitive voltage-gated K^+^
LF	low frequency
LV	left ventricular
MMP	matrix metaloproteinase
NADH	nicotinamide adenine dinucleotide, reduced form
NF-κB	activate nuclear factor kappa B
NLRP3	NOD-like receptor pyrin domain-containing protein 3
NSVT	non-sustained ventricular tachycardia
NTP	negative thoracic pressure
OR	odds ratio
OSA	obstructive sleep apnea
RAAS	renin–angiotensin–aldosterone system
RAGE	receptor for advanced glycation end products
REM	rapid eye movement
ROS	reactive oxygen species
PRV	pulse rate variability
SAS	sleep apnea syndrome
SASP	senescence-associated secretory phenotype
SCD	sudden cardiac death
SDB	sleep-disordered breathing
sST2	soluble suppression of tumorigenicity-2
TGF-β	transforming growth factor beta
TNF-α	tumor necrosis factor-α
VCAM-1	vascular cell adhesion molecule-1
VT	ventricular tachycardia

## References

[1] Yeghiazarians Y., Jneid H., Tietjens J., Redline S., Brown D., El-Sherif N., Mehra R., Bozkurt B., Ndumele C.E., Somers V. (2021). Obstructive sleep apnea and cardiovascular disease: A scientific statement from the American Heart Association. Circulation.

[2] Kapur V., Auckley D., Chowdhuri S., Kuhlmann D., Mehra R., Ramar K., Harrod C. (2017). Clinical practice guideline for diagnostic testing for adult obstructive sleep apnea: An american academy of sleep medicine clinical practice guideline. J. Clin. Sleep Med..

[3] Gottlieb D.J., Yenokyan G., Newman A.B., O’Connor G.T., Punjabi N.M., Quan S.F., Redline S., Resnick H.E., Tong E.K., Diener-West M. (2010). Prospective study of obstructive sleep apnea and incident coronary heart disease and heart failure: The sleep heart health study. Circulation.

[4] Yaggi H.K., Concato J., Kernan W.N., Lichtman J.H., Brass L.M., Mohsenin V. (2005). Obstructive sleep apnea as a risk factor for stroke and death. N. Engl. J. Med..

[5] Javaheri S., Barbe F., Campos-Rodriguez F., Dempsey J.A., Khayat R., Javaheri S., Malhotra A., Martinez-Garcia M.A., Mehra R., Pack A.I. (2017). Sleep apnea: Types, mechanisms, and clinical cardiovascular consequences. J. Am. Coll. Cardiol..

[6] Guilleminault C., Connolly S.J., Winkle R.A. (1983). Cardiac arrhythmia and conduction disturbances during sleep in 400 patients with sleep apnea syndrome. Am. J. Cardiol..

[7] Wolk R., Kara T., Somers V.K. (2003). Sleep-disordered breathing and cardiovascular disease. Circulation.

[8] Chang T.Y., Liao J.N., Chao T.F., Vicera J.J., Lin C.Y., Tuan T.C., Lin Y.J., Chang S.L., Lo L.W., Hu Y.F. (2018). Oral anticoagulant use for stroke prevention in atrial fibrillation patients with difficult scenarios. Int. J. Cardiol. Heart Vasc..

[9] Stevenson I.H., Teichtahl H., Cunnington D., Ciavarella S., Gordon I., Kalman J.M. (2008). Prevalence of sleep disordered breathing in paroxysmal and persistent atrial fibrillation patients with normal left ventricular function. Eur. Heart J..

[10] Becker H.F., Koehler U., Stammnitz A., Peter J.H. (1998). Heart block in patients with sleep apnea. Thorax.

[11] Gami A.S., Howard D.E., Olson E.J., Somers V.K. (2005). Day-night pattern of sudden death in obstructive sleep apnea. N. Engl. J. Med..

[12] Mehra R., Benjamin E.J., Shahar E., Gottlieb D.J., Nawabit R., Kirchner H.L., Sahadevan J., Redline S. (2006). Association of nocturnal arrhythmias with sleep-disordered breathing: The sleep heart health study. Am. J. Respir. Crit. Care Med..

[13] Tanigawa T., Yamagishi K., Sakurai S., Muraki I., Noda H., Shimamoto T., Iso H. (2006). Arterial oxygen desaturation during sleep and atrial fibrillation. Heart.

[14] Gami A.S., Hodge D.O., Herges R.M., Olson E.J., Nykodym J., Kara T., Somers V.K. (2007). Obstructive sleep apnea, obesity, and the risk of incident atrial fibrillation. J. Am. Coll. Cardiol..

[15] Garrigue S., Pépin J.L., Defaye P., Murgatroyd F., Poezevara Y., Clémenty J., Lévy P. (2007). High prevalence of sleep apnea syndrome in patients with long-term pacing: The European multicenter polysomnographic study. Circulation.

[16] Monahan K., Storfer-Isser A., Mehra R., Shahar E., Mittleman M., Rottman J., Punjabi N., Sanders M., Quan S.F., Resnick H. (2009). Triggering of nocturnal arrhythmias by sleep-disordered breathing events. J. Am. Coll. Cardiol..

[17] Gami A.S., Olson E.J., Shen W.K., Wright R.S., Ballman K.V., Hodge D.O., Herges R.M., Howard D.E., Somers V.K. (2013). Obstructive sleep apnea and the risk of sudden cardiac death: A longitudinal study of 10,701 adults. J. Am. Coll. Cardiol..

[18] Holmqvist F., Guan N., Zhu Z., Kowey P.R., Allen L.A., Fonarow G.C., Hylek E.M., Mahaffey K.W., Freeman J.V., Chang P. (2015). Impact of obstructive sleep apnea and continuous positive airway pressure therapy on outcomes in patients with atrial fibrillation—Results from the outcomes registry for better informed treatment of atrial fibrillation (ORBIT-AF). Am. Heart J..

[19] Cadby G., McArdle N., Briffa T., Hillman D.R., Simpson L., Knuiman M., Hung J. (2015). Severity of OSA is an independent predictor of incident atrial fibrillation hospitalization in a large sleep-clinic cohort. Chest.

[20] Selim B.J., Koo B.B., Qin L., Jeon S., Won C., Redeker N.S., Lampert R.J., Concato J.P., Bravata D.M., Ferguson J. (2016). The association between nocturnal cardiac arrhythmias and sleep-disordered breathing: The DREAM study. J. Clin. Sleep Med..

[21] Reshetnik A., Puppe S., Bonnemeier H. (2019). Central sleep apnea and arrhythmogenesis after myocardial infarction: The CESAAR study. Front. Cardiovasc. Med..

[22] Saleeb-Mousa J., Nathanael D., Coney A.M., Kalla M., Brain K.L., Holmes A.P. (2023). Mechanisms of atrial fibrillation in obstructive sleep apnoea. Cells.

[23] Wallin B.G., Delius W., Sundlof G. (1974). Human muscle nerve sympathetic activity in cardiac arrhythmias. Scand. J. Clin. Lab. Investig..

[24] Kasai T., Bradley T.D. (2011). Obstructive sleep apnea and heart failure: Pathophysiologic and therapeutic implications. J. Am. Coll. Cardiol..

[25] Parish J.M., Somers V.K. (2004). Obstructive sleep apnea and cardiovascular disease. Mayo Clin. Proc..

[26] Linz D., Schotten U., Neuberger H.R., Böhm M., Wirth K. (2011). Negative tracheal pressure during obstructive respiratory events promotes atrial fibrillation by vagal activation. Heart Rhythm.

[27] Orban M., Bruce C.J., Pressman G.S., Leinveber P., Romero-Corral A., Korinek J., Konecny T., Villarraga H.R., Kara T., Caples S.M. (2008). Dynamic changes of left ventricular performance and left atrial volume induced by the mueller maneuver in healthy young adults and implications for obstructive sleep apnea, atrial fibrillaion and heart failure. Am. J. Cardiol..

[28] Linz D., Schotten U., Neuberger H.R., Böhm M., Wirth K. (2011). Combined blockade of early and late activated atrial potassium currents suppresses atrial fibrillation in a pig model of obstructive apnea. Heart Rhythm.

[29] Linz D., Mahfoud F., Schotten U. (2012). Renal sympathetic denervation suppresses postapneic blood pressure rises and atrial fibrillation in a model for sleep apnea. Hypertension.

[30] Rogers R.M., Spear J.F., Moore E.N., Horowitz L.H., Sonne J.E. (1973). Vulnerability of canine ventricle to fibrillation during hypoxia and respiratory acidosis. Chest.

[31] Loke Y.K., Brown J.W., Kwok C.S., Niruban A., Myint P.K. (2012). Association of obstructive sleep apnea with risk of serious cardiovascular events: A systematic review and meta-analysis. Circ. Cardiovasc. Qual. Outcomes.

[32] Solin P., Bergin P., Richardson M., Kaye D.M., Walters E.H., Naughton M.T. (1999). Influence of pulmonary capillary wedge pressure on central apnea in heart failure. Circulation.

[33] Braghieri L., Younis A., Tabaja C., Santangeli P., Taigen T., Rickard J., Callahan T., Martin D.O., Nakhla S., Kanj M. (2025). Impact of obstructive sleep apnea on arrhythmia and quality-of-life outcomes after catheter ablation of atrial fibrillation. J. Am. Heart Assoc..

[34] Young T., Palta M., Dempsey J., Skatrud J., Weber S., Badr S. (1993). The occurrence of sleep-disordered breathing among middle-aged adults. N. Engl. J. Med..

[35] Somers V.K., Dyken M.E., Skinner J.L. (1993). Autonomic and hemodynamic responses and interactions during the Mueller maneuver in humans. J. Auton. Nerv. Syst..

[36] Otto M.E., Belohlavek M., Romero-Corral A., Gami A.S., Gilman G., Svatikova A., Amin R.S., Lopez-Jimenez F., Khandheria B.K., Somers V.K. (2007). Comparison of cardiac structural and functional changes in obese otherwise healthy adults with versus without obstructive sleep apnea. Am. J. Cardiol..

[37] Romero-Corral A., Somers V.K., Pellikka P.A., Olson E.J., Bailey K.R., Korinek J., Orban M., Sierra-Johnson J., Kato M., Amin R.S. (2007). Decreased right and left ventricular myocardial performance in obstructive sleep apnea. Chest.

[38] Haissaguerre M., Jais P., Shah D.C., Takahashi A., Hocini M., Quiniou G., Le Mouroux A., Le Metayer P., Clementy J. (1998). Spontaneous initiation of atrial fibrillation by ectopic beats originating in the pulmonary veins. N. Engl. J. Med..

[39] Pejovic S., Vgontzas A.N., Fernandez-Mendoza J.A., Fan H., Lin Y., Karataraki M., Bixler E.O. (2024). Obstructive sleep apnea comorbid with insomnia symptoms and objective short sleep duration is associated with clinical and preclinical cardiometabolic risk factors: Clinical implications. Sleep Med..

[40] Lanfranchi P.A., Braghiroli A., Bosimini E., Mazzuero G., Colombo R., Donner C.F., Giannuzzi P. (1999). Prognostic value of nocturnal Cheyne-Stokes respiration in chronic heart failure. Circulation.

[41] Veasey S.C., Rosen I.M. (2019). Obstructive sleep apnea in adults. N. Engl. J. Med..

[42] Shahar E., Whitney C.W., Redline S., Lee E.T., Newman A.B., Nieto F.J., O’Connor G.T., Boland L.L., Schwartz J.E., Samet J.M. (2001). Sleep-disordered breathing and cardiovascular disease: Cross-sectional results of the Sleep Heart Health Study. Am. J. Respir. Crit. Care Med..

[43] Bradley T.D., Floras J.S. (2003). Sleep apnea and heart failure: Part II: Central sleep apnea. Circulation.

[44] Javaheri S., Parker T.J., Liming J.D., Corbett W.S., Nishiyama H., Wexler L., Roselle G.A. (1998). Sleep apnea in 81 ambulatory male patients with stable heart failure. Types and their prevalences, consequences, and presentations. Circulation.

[45] Schotten U., Verheule S., Kirchhof P., Goette A. (2011). Pathophysiological mechanisms of atrial fibrillation: A translational appraisal. Physiol. Rev..

[46] Nalliah C.J., Wong G.R., Lee G., Voskoboinik A., Kee K., Goldin J., Watts T., Linz D., Wirth D., Parameswaran R. (2021). Sleep apnoea has a dosedependent effect on atrial remodelling in paroxysmal but not persistent atrial fibrillation: A high-density mapping study. EP Europace.

[47] Affas Z., Affas S., Tabbaa K. (2022). Continuous positive airway pressure reduces the incidence of atrial fibrillation in patients with obstructive sleep apnea: A meta-analysis and systematic review. Spartan Med. Res. J..

[48] Lundetræ R.S., Lehmann S., Saxvig I.W., Saeed S., Gislason T., Bjorvatn B. (2025). Severity of obstructive sleep apnea is related to C-reactive protein levels: The influence of comorbidities and self-reported sleep duration. Sleep Med..

[49] Dewland T.A., Vittinghoff E., Mandyam M.C. (2013). Atrial ectopy as a predictor of incident atrial fibrillation: A cohort study. Ann. Intern. Med..

[50] Oldenburg O., Bitter T., Wiemer M., Langer C., Horstkotte D. (2009). Pulmonary capillary wedge pressure and pulmonary arterial pressure in heart failure patients with sleep disordered breathing. Sleep Med..

[51] Straus S.M., Kors J.A., De Bruin M.L. (2006). Prolonged QTc interval and risk of sudden cardiac death in a population of older adults. J. Am. Coll. Cardiol..

[52] Javaheri S. (1999). A mechanism of central sleep apnea in patients with heart failure. N. Engl. J. Med..

[53] Koshino Y., Villarraga H.R., Orban M., Bruce C.J., Pressman G.S., Leinveber P., Saleh H.K., Konecny T., Kara T., Somers V.K. (2010). Changes in left and right ventricular mechanics during the Mueller maneuver in healthy adults: A possible mechanism for abnormal cardiac function in patients with obstructive sleep apnea. Circ. Cardiovasc. Imaging.

[54] Jongnarangsin K., Chugh A., Good E., Mukerji S., Dey S., Crawford T., Sarrazin J.F., Kuhne M., Chalfoun N., Wells D. (2008). Body mass index, obstructive sleep apnea, and outcomes of catheter ablation of atrial fibrillation. J. Cardiovasc. Electrophysiol..

[55] Drager L.F., Bortolotto L.A., Figueiredo A.C., Silva B.C., Krieger E.M., Lorenzi-Filho G. (2007). Obstructive sleep apnea, hypertension, and their interaction on arterial stiffness and heart remodeling. Chest.

[56] Cioffi G., Russo T.E., Stefenelli C. (2010). Severe obstructive sleep apnea elicits concentric left ventricular geometry. J. Hypertens..

[57] Lisan Q., Van Sloten T., Marques Vidal P., Haba R.J., Heinzer R., Empana J.P. (2019). Association of positive airway pressure prescription with mortality in patients with obesity and severe obstructive sleep apnea: The Sleep Heart Health Study. JAMA Otolaryngol. Head. Neck Surg..

[58] Shepard J.W., Garrison M.W., Grither D.A., Evans R., Schweitzer P.K. (1985). Relationship of ventricular ectopy to nocturnal oxygen desaturation in patients with chronic obstructive pulmonary disease. Am. J. Med..

[59] Zhang L., Hou Y., Sunny S.P. (2015). Obstructive Sleep Apnoea and Atrial Fibrillation. Arrhythmia Electrophysiol. Rev..

[60] Rossi V.A., Stradling J.R., Kohler M. (2013). Effects of obstructive sleep apnoea on heart rhythm. Eur. Respir. J..

[61] Peppard P.E., Szklo-Coxe M., Hla K.M., Young T. (2006). Longitudinal association of sleep-related breathing disorder and depression. Arch. Intern. Med..

[62] Kendzerska T., Gershon A.S., Hawker G., Tomlinson G., Leung R.S. (2014). Obstructive sleep apnea and incident diabetes: A historical cohort study. Am. J. Respir. Crit. Care Med..

[63] Toischer K., Rokita A.G., Unsöld B., Zhu W., Kararigas G., Sossalla S., Reuter S.P., Becker A., Teucher N., Seidler T. (2010). Differential cardiac remodeling in preload versus afterload. Circulation.

[64] Erickson J.R., Joiner M.L., Guan X., Kutschke W., Yang J., Oddis C.V., Bartlett R.K., Lowe J.S., O’Donnell S.E., Aykin-Burns N. (2008). A dynamic pathway for calcium-independent activation of CaMKII by methionine oxidation. Cell.

[65] Wagner S., Ruff H.M., Weber S.L., Bellmann S., Sowa T., Schulte T., Anderson M.E., Grandi E., Bers D.M., Backs J. (2011). Reactive oxygen species-activated Ca/calmodulin kinase IIδ is required for late I(Na) augmentation leading to cellular Na and Ca overload. Circ. Res..

[66] Dybkova N., Wagner S., Backs J., Hund T.J., Mohler P.J., Sowa T., Nikolaev V.O., Maier L.S. (2014). Tubulin polymerization disrupts cardiac β-adrenergic regulation of late INa. Cardiovasc. Res..

[67] Quan S.F., Howard B.V., Iber C., Kiley J.P., Nieto F.J., O’Connor G.T., Rapoport D.M., Redline S., Robbins J., Samet J.M. (1997). The Sleep Heart Health Study: Design, rationale, and methods. Sleep.

[68] Bixler E.O., Vgontzas A.N., Lin H.M., Have T.T., Rein J., Vela-Bueno A., Kales A. (2001). Prevalence of sleep-disordered breathing in women: Effects of gender. Am. J. Respir. Crit. Care Med..

[69] Peppard P.E., Young T., Barnet J.H., Palta M., Hagen E.W., Hla K.M. (2013). Increased prevalence of sleep-disordered breathing in adults. Am. J. Epidemiol..

[70] Tufik S., Santos-Silva R., Taddei J.A., Bittencourt L.R. (2010). Obstructive sleep apnea syndrome in the Sao Paulo Epidemiologic Sleep Study. Sleep Med..

[71] De Jong A.M., Maass A.H., Oberdorf-Maass S.U., Van Veldhuisen D.J., Van Gilst W.H., Van Gelder I.C. (2011). Mechanisms of atrial structural changes caused by stretch occurring before and during early atrial fibrillation. Cardiovasc. Res..

[72] Ryan C.M., Juvet S., Leung R., Bradley T.D. (2008). Timing of nocturnal ventricular ectopy in heart failure patients with sleep apnea. Chest.

[73] Tavares L., Lador A., Valderrábano M. (2021). Sleep apnea and atrial fibrillation: Role of the cardiac autonomic nervous system. Methodist. Debakey Cardiovasc. J..

[74] Grassi G., Seravalle G., Bertinieri G., Mancia G. (2003). Behaviour of the adrenergic cardiovascular drive in atrial fibrillation and cardiac arrhythmias. Acta Physiol. Scand..

[75] Kholdani C., Fares W.H., Mohsenin V. (2015). Pulmonary hypertension in obstructive sleep apnea: Is it clinically significant? A critical analysis of the association and pathophysiology. Pulm. Circ..

[76] Holt A., Bjerre J., Zareini B., Koch H., Tønnesen P., Gislason G.H., Nielsen O.W., Schou M., Lamberts M. (2018). Sleep apnea, the risk of developing heart failure, and potential benefits of continuous positive airway pressure (CPAP) therapy. J. Am. Heart Assoc..

[77] Javaheri S., Corbett W.S. (1998). Association of low PaCO2 with central sleep apnea and ventricular arrhythmias in ambulatory patients with stable heart failure. Ann. Intern. Med..

[78] Condos W.R., Latham R.D., Hoadley S.D., Pasipoularides A. (1987). Hemodynamics of the Mueller maneuver in man: Right and left heart micromanometry and Doppler echocardiography. Circulation.

[79] Stevenson I.H., Roberts-Thomson K.C., Kistler P.M., Edwards G.A., Spence S., Sanders P., Kalman J.M. (2010). Atrial electrophysiology is altered by acute hypercapnia but not hypoxemia: Implications for promotion of atrial fibrillation in pulmonary disease and sleep apnea. Heart Rhythm.

[80] De Daly M.B., Scott M.J. (1958). The effects of stimulation of the carotid body chemoreceptors on heart rate in the dog. J. Physiol..

[81] Daly M.D., Scott M.J. (1963). The cardiovascular responses to stimulation of the carotid body chemoreceptors in the dog. J. Physiol..

[82] Peng Y.J., Yuan G., Ramakrishnan D., Sharma S.D., Bosh-Marce M., Kumar G.K., Semenza G.L., Prabhakar N.R. (2006). Heterozygous HIF-1α deficiency impairs carotid body-mediated systemic responses and reactive oxygen species generation in mice exposed to intermittent hypoxia. J. Physiol..

[83] Duran J., Esnaola S., Rubio R., Iztueta A. (2001). Obstructive sleep apnea-hypopnea and related clinical features in a population-based sample of subjects aged 30 to 70 yr. Am. J. Respir. Crit. Care Med..

[84] Berry R.B., Budhiraja R., Gottlieb D.J., Gozal D., Iber C., Kapur V.K., Marcus C.L., Mehra R., Parthasarathy S., Quan S.F. (2012). Rules for scoring respiratory events in sleep: Update of the 2007 AASM manual for the scoring of sleep and associated events. J. Clin. Sleep Med..

[85] Iturriaga R., Diaz H.S. (2025). Reactive Oxidative Species in Carotid Body Chemoreception: Their Role in Oxygen Sensing and Cardiorespiratory Alterations Induced by Chronic Intermittent Hypoxia. Antioxidants.

[86] Kanagala R., Murali N.S., Friedman P.A., Ammash N.M., Gersh B.J., Ballman K.V., Shamsuzzaman A.S.M., Somers V.K. (2003). Obstructive sleep apnea and the recurrence of atrial fibrillation. Circulation.

[87] Mody P., Rukhadze I., Kubin L. (2011). Rats subjected to chronic-intermittent hypoxia have increased density of noradrenergic terminals in the trigeminal sensory and motor nuclei. Neurosci. Lett..

[88] Rukhadze I., Fenik V.B., Benincasa K.E. (2010). Chronic intermittent hypoxia alters density of aminergic terminals and receptors in the hypoglossal motor nucleus. Am. J. Respir. Crit. Care Med..

[89] Souvannakitti D., Kumar G.K., Fox A., Prabhakar N.R. (2009). Contrasting effects of intermittent and continuous hypoxia on low O_2_ evoked catecholamine secretion from neonatal rat chromaffin cells. Adv. Exp. Med. Biol..

[90] Hla K.M., Young T.B., Bidwell T., Palta M., Skatrud J.B., Dempsey J. (1994). Sleep apnea and hypertension. A population-based study. Ann. Intern. Med..

[91] Shen M.J., Zipes D.P. (2014). Role of the autonomic nervous system in modulating cardiac arrhythmias. Circ. Res..

[92] Ng G.A., Brack K.E., Coote J.H. (2001). Effects of direct sympathetic and vagus nerve stimulation on the physiology of the whole heart–a novel model of isolated Langendorff perfused rabbit heart with intact dual autonomic innervation. Exp. Physiol..

[93] Jo J.A., Blasi A., Valladares E., Juarez R., Baydur A., Khoo M.C.K. (2005). Determinants of heart rate variability in obstructive sleep apnea syndrome during wakefulness and sleep. Am. J. Physiol. Heart Circ. Physiol..

[94] Hall M.J., Ando S., Floras J.S., Bradley T.D. (1998). Magnitude and time course of hemodynamic responses to Mueller maneuvers in patients with congestive heart failure. J. Appl. Physiol..

[95] Franz M.R., Bode F. (2003). Mechano-electrical feedback underlying arrhythmias: The atrial fibrillation case. Prog. Biophys. Mol. Biol..

[96] Leung R.S. (2009). Sleep-disordered breathing: Autonomic mechanisms and arrhythmias. Prog. Cardiovasc. Dis..

[97] Guilleminault C., Connolly S., Winkle R., Melvin K., Tilkian A. (1984). Cyclic variation of the heart rate in sleep apnea syndrome. Mechanisms, and usefulness of 24 h electrocardiography as a screening technique. Lancet.

[98] Hayano J., Watanabe E., Saito Y., Sasaki F., Fujimoto K., Nomiyama T., Kawai K., Kodama I., Sakakibara H. (2011). Screening for obstructive sleep apnea by cyclic variation of heart rate. Circ. Arrhythm. Electrophysiol..

[99] Chung M.K., Martin D.O., Sprecher D., Wazni O., Kanderian A., Carnes C.A., Bauer J.A., Tchou P.J., Niebauer M.J., Natale A. (2001). C-reactive protein elevation in patients with atrial arrhythmias: Inflammatory mechanisms and persistence of atrial fibrillation. Circulation.

[100] Sahrai S., Romarheim A., Mancia G., West Saxvig I., Gulati S., Lehmann S., Bjorvatn B. (2022). Characteristics of hypertension and arterial stiffness in obstructive sleep apnea: A Scandinavian experience from a prospective study of 6408 normotensive and hypertensive patients. J. Clin. Hypertens..

[101] Black N., D’Souza A., Wang Y., Piggins H., Dobrzynski H., Morris G., Boyett M.R. (2019). Circadian rhythm of cardiac electrophysiology, arrhythmogenesis, and the underlying mechanisms. Heart Rhythm.

[102] DeMartino T., El Ghoul R., Wang L. (2016). Oxidative stress and inflammation differentially elevated in objective versus habitual subjective reduced sleep duration in obstructive sleep apnea. Sleep.

[103] Unnikrishnan D., Jun J., Polotsky V. (2015). Inflammation in sleep apnea: An update. Rev. Endocr. Metab. Disord..

[104] Geovanini G.R., Wang R., Weng J., Tracy R., Jenny N.S., Goldberger A.L., Costa M.D., Liu Y., Libby P., Redline S. (2018). Elevations in neutrophils with obstructive sleep apnea: The multi-ethnic study of atherosclerosis (MESA). Int. J. Cardiol..

[105] Nadeem R., Molnar J., Madbouly E.M., Nida M., Aggarwal S., Sajid H., Naseem J., Loomba R. (2013). Serum inflammatory markers in obstructive sleep apnea: A meta-analysis. J. Clin. Sleep Med..

[106] (2005). 2004 Canadian Cardiovascular Society Consensus Conference: Atrial fibrillation. Can. J. Cardiol..

[107] Gami A.S., Pressman G., Caples S.M., Kanagala R., Gard J.J., Davison D.E., Malouf J.F., Ammash N.M., Friedman P.A., Somers V.K. (2004). Association of atrial fibrillation and obstructive sleep apnea. Circulation.

[108] Oldenburg O., Lamp B., Faber L., Teschler H., Horstkotte D., Töpfer V. (2007). Sleep-disordered breathing in patients with symptomatic heart failure: A contemporary study of prevalence in and characteristics of 700 patients. Eur. J. Heart Fail..

[109] Ucar Y., Yonar A. (2025). Evaluation of inflammatory markers in obstructive sleep apnea syndrome. Sci. Prog..

[110] Leung R.S., Floras J.S., Bradley T.D. (2006). Respiratory modulation of the autonomic nervous system during Cheyne-Stokes respiration. Can. J. Physiol. Pharmacol..

[111] Benjamin E.J., Wolf P.A., D’Agostino R.B., Silbershatz H., Kannel W.B., Levy D. (1998). Impact of atrial fibrillation on the risk of death: The Framingham Heart Study. Circulation.

[112] Chen X., Wang R., Zee P., Lutsey P.L., Javaheri S., Alcántara C., Jackson C.L., Williams M.A., Redline S. (2015). Racial/ethnic differences in sleep disturbances: The multi-ethnic study of atherosclerosis (MESA). Sleep.

[113] Zhao J., Xu W., Yun F., Zhao H., Li W., Gong Y., Yuan Y., Yan S., Zhang S., Ding X. (2014). Chronic obstructive sleep apnea causes atrial remodeling in canines: Mechanisms and implications. Basic Res. Cardiol..

[114] Miao S., Yang Y., Li R., Yin L., Zhang K., Cheng L., Xu X., Wang W., Zhao Z., Li G. (2020). The potential effects of Aliskiren on atrial remodeling induced by chronic intermittent hypoxia in rats. Drug Des. Dev. Ther..

[115] Yang Y., Liu Y., Ma C., Li R., Yang Q., Zhang K., Cheng L., Yuan M., Zhang Y., Zhao Z. (2022). Improving effects of eplerenone on atrial remodeling induced by chronic intermittent hypoxia in rats. Cardiovasc. Pathol..

[116] Zhang K., Ma Z., Song C., Duan X., Yang Y., Li G. (2020). Role of ion channels in chronic intermittent hypoxia-induced atrial remodeling in rats. Life Sci..

[117] Grant A.O. (2009). Cardiac ion channels. Circ. Arrhythm. Electrophysiol..

[118] Del Rio R., Andrade D.C., Lucero C., Arias P., Iturriaga R. (2016). carotid body ablation abrogates hypertension and autonomic alterations induced by intermittent hypoxia in rats. Hypertension.

[119] Bode D., Pronto J.R.D., Schiattarella G.G., Voigt N. (2024). Metabolic remodelling in atrial fibrillation: Manifestations, mechanisms and clinical implications. Nat. Rev. Cardiol..

[120] Zhang K., Zhao L., Ma Z., Wang W., Li X., Zhang Y., Yuan M., Liang X., Li G. (2018). Doxycycline attenuates atrial remodeling by interfering with microRNA-21 and downstream phosphatase and tensin homolog (PTEN)/phosphoinositide 3-kinase (PI3K) signaling pathway. Med. Sci. Monit..

[121] Ramos P., Rubies C., Torres M., Batlle M., Farre R., Brugada J., Montserrat J., Almendros I., Mont L. (2014). Atrial fibrosis in a chronic murine model of obstructive sleep apnea: Mechanisms and prevention by mesenchymal stem cells. Respir. Res..

[122] Itani O., Jike M., Watanabe N., Kaneita Y. (2017). Short sleep duration and health outcomes: A systematic review, meta-analysis, and meta-regression. Sleep Med..

[123] Azarbarzin A., Sands S.A., Stone K.L., Taranto-Montemurro L., Messineo L., Terrill P.I., Ancoli-Israel S., Ensrud K., Purcell S., White D.P. (2019). The hypoxic burden of sleep apnoea predicts cardiovascular disease-related mortality: The osteoporotic fractures in men study and the sleep heart health study. Eur. Heart J..

[124] Sheng X., Scherlag B.J., Yu L., Li S., Ali R., Zhang Y., Fu G., Nakagawa H., Jackman W., Lazzara R. (2011). Prevention and reversal of atrial fibrillation inducibility and autonomic remodeling by low-level vagosympathetic nerve stimulation. J. Am. Coll. Cardiol..

[125] Lim D.C., Brady D.C., Po P., Chuang L.P., Marcondes L., Kim E., Keenan B., Guo X., Maislin G., Galante R. (2015). Simulating obstructive sleep apnea patients’ oxygenation characteristics into a mouse model of cyclical intermittent hypoxia. J. Appl. Physiol..

[126] Zhang K., Ma Z., Wang W., Liu R., Zhang Y., Yuan M., Li G. (2018). Beneficial effects of tolvaptan on atrial remodeling induced by chronic intermittent hypoxia in rats. Cardiovasc. Ther..

[127] Linz D., Hohl M., Khoshkish S., Mahfoud F., Ukena C., Neuberger H.R., Wirth K., Böhm M. (2016). Low-level but not high-level baroreceptor stimulation inhibits atrial fibrillation in a pig model of sleep apnea. J. Cardiovasc. Electrophysiol..

[128] Guo Y., Xiaokereti J., Meng Q., Cao G., Sun H., Zhou X., Zhang L., Tang B. (2020). Low-level vagus nerve stimulation reverses obstructive sleep apnea-related atrial fibrillation by ameliorating sympathetic hyperactivity and atrial myocyte injury. Front. Physiol..

[129] Lubis A.C., Sarastri Y., Cut A.A., Masyab N., Fauziyah H. (2025). obstructive sleep apnea in patients with heart failure and atrial fibrillation. Sumat. Med. J..

[130] Tao L., Wang L., Yang X., Jiang X., Hua F. (2019). Recombinant human glucagon-like peptide-1 protects against chronic intermittent hypoxia by improving myocardial energy metabolism and mitochondrial biogenesis. Mol. Cell Endocrinol..

[131] Chen Y.L., Chen Y.C., Wang H.T., Chang Y.T., Fang Y.N., Hsueh S., Liu W., Lin P., Hsu P., Su M. (2022). the impact of intermittent hypoxemia on left atrial remodeling in patients with obstructive sleep apnea syndrome. Life.

[132] Chugh S.S., Havmoeller R., Narayanan K., Singh D., Rienstra M., Benjamin E.J., Gillum R.F., Kim Y.H., McAnulty J.H., Zheng Z.J. (2014). Worldwide epidemiology of atrial fibrillation: A Global Burden of Disease 2010 Study. Circulation.

[133] Nanduri J., Vaddi D.R., Khan S.A., Wang N., Makerenko V., Prabhakar N.R. (2013). Xanthine oxidase mediates hypoxia-inducible factor-2α degradation by intermittent hypoxia. PLoS ONE.

[134] Eisele H.J., Markart P., Schulz R. (2015). Obstructive sleep apnea, oxidative stress, and cardiovascular disease: Evidence from human studies. Oxid. Med. Cell Longev..

[135] McDonagh T.A., Metra M., Adamo M., Gardner R.S., Baumbach A., Böhm M., Burri H., Butler J., Čelutkienė J., Chioncel O. (2021). ESC Guidelines for the diagnosis and treatment of acute and chronic heart failure. Eur. Heart J..

[136] Tse G., Yan B.P., Chan Y.W., Tian X.Y., Huang Y. (2016). Reactive oxygen species, endoplasmic reticulum stress and mitochondrial dysfunction: The link with cardiac arrhythmogenesis. Front. Physiol..

[137] Mastino P., Rosati D., de Soccio G., Romeo M., Pentangelo D., Venarubea S., Fiore M., Meliante P.G., Petrella C., Barbato C. (2023). Oxidative stress in obstructive sleep apnea syndrome: Putative pathways to hearing system impairment. Antioxidants.

[138] Nanduri J., Wang N., Yuan G., Khan S.A., Souvannakitti D., Peng Y.J., Kumar G.K., Garcia J.A., Prabhakar N.R. (2009). Intermittent hypoxia degrades HIF-2alpha via calpains resulting in oxidative stress: Implications for recurrent apnea-induced morbidities. Proc. Natl. Acad. Sci. USA.

[139] Lavie L. (2010). Oxidative stress and inflammation in OSA. Eur. Respir. Monogr..

[140] May A.M., Mehra R. (2014). Obstructive sleep apnea: Role of intermittent hypoxia and inflammation. Semin. Respir. Crit. Care Med..

[141] Karamanlı H., Özol D., Ugur K.S., Yildirim Z., Armutcu F., Bozkurt B., Yigitoglu R. (2014). Influence of CPAP treatment on airway and systemic inflammation in OSAS patients. Sleep Breath..

[142] Murri M., García-Delgado R., Alcázar-Ramírez J., Fernandez-Ramos A., Alcaide J., Cardona F., Tinahones F.J. (2011). Effect of CPAP on oxidative stress and circulating progenitor cell levels in sleep patients with apnea-hypopnea syndrome. Respir. Care.

[143] Chen L., Zhang J., Gan T.X., Chen-Izu Y., Hasday J.D., Karmazyn M., Balke C.W., Scharf M. (2008). Left ventricular dysfunction and associated cellular injury in rats exposed to chronic intermittent hypoxia. J. Appl. Physiol..

[144] Liu J.N., Zhang J.X., Lu G., Qiu Y., Yang D., Yin G.Y., Zhang X.L. (2010). The effect of oxidative stress in myocardial cell injury in mice exposed to chronic intermittent hypoxia. Chin. Med. J..

[145] Somers V.K., Dyken M.E., Clary M.P., Abboud F.M. (1995). Sympathetic neural mechanisms in obstructive sleep apnea. J. Clin. Investig..

[146] Chen L., Einbinder E., Zhang Q., Hasday J., Balke C.W., Scharf S.M. (2005). Oxidative stress and left ventricular function with chronic intermittent hypoxia in rats. Am. J. Respir. Crit. Care Med..

[147] Jeong E.M., Liu M., Sturdy M., Gao G., Varghese S.T., Sovari A.A., Dudley S.C. (2012). Metabolic stress, reactive oxygen species, and arrhythmia. J. Mol. Cell Cardiol..

[148] Tirlapur V.G. (1985). Relationship of ventricular ectopy to nocturnal oxygen desaturation in patients with chronic obstructive pulmonary disease. Am. J. Med..

[149] Cortassa S., Aon M.A., Marbán E., Winslow R.L., O’Rourke B. (2003). An integrated model of cardiac mitochondrial energy metabolism and calcium dynamics. Biophys. J..

[150] Murnaghan M.F. (1975). The effect of anoxia on the ventricular fibrillation threshold in the rabbit isolated heart. Br. J. Pharmacol..

[151] Brown D.A., O’Rourke B. (2010). Cardiac mitochondria and arrhythmias. Cardiovasc. Res..

[152] Narkiewicz K., van de Borne P.J., Cooley R.L., Dyken M.E., Somers V.K. (1998). Sympathetic activity in obese subjects with and without obstructive sleep apnea. Circulation.

[153] Kumar G.K., Rai V., Sharma S.D., Ramakrishnan D.P., Peng Y.J., Souvannakitti D., Prabhakar N.R. (2006). Chronic intermittent hypoxia induces hypoxia-evoked catecholamine efflux in adult rat adrenal medulla via oxidative stress. J. Physiol..

[154] Froese N., Szaroszyk M., Galuppo P., Visker J.R., Werlein C., Korf-Klingebiel M., Berliner D., Reboll M.R., Hamouche R., Gegel S. (2024). Hypoxia attenuates pressure overload-induced heart failure. J. Am. Heart Assoc..

[155] Wever-Pinzon O., Drakos S.G., McKellar S.H., Horne B.D., Caine W.T., Kfoury A.G., Li D.Y., Fang J.C., Stehlik J., Selzman C.H. (2016). Cardiac recovery during long-term left ventricular assist device support. J. Am. Coll. Cardiol..

[156] Park A.M., Suzuki Y.J. (2007). Effects of intermittent hypoxia on oxidative stress-induced myocardial damage in mice. J. Appl. Physiol..

[157] Akar F.G., Maack C. (2023). Top Stories: Mitochondrial origin of inherited cardiac arrhythmias. Heart Rhythm.

[158] Fei J., Demillard L.J., Ren J. (2022). Reactive oxygen species in cardiovascular diseases: An update. Explor. Med..

[159] Møller D.S., Lind P., Strunge B., Pedersen E.B. (2003). Abnormal vasoactive hormones and 24-hour blood pressure in obstructive sleep apnea. Am. J. Hypertens..

[160] Mancuso M., Bonanni E., LoGerfo A., Orsucci D., Maestri M., Chico L., DiCoscio E., Fabbrini M., Siciliano G., Murri L. (2012). Oxidative stress biomarkers in patients with untreated obstructive sleep apnea syndrome. Sleep Med..

[161] Nalliah C.J., Wong G.R., Lee G., Voskoboinik A., Kee K., Goldin J., Watts T., Linz D., Parameswaran R., Sugumar H. (2022). Impact of CPAP on the atrial fibrillation substrate in obstructive sleep apnea: The SLEEP-AF study. JACC Clin. Electrophysiol..

[162] McQueen D.S., Ungar A. (1969). The direct and crossed vagal components of the reflex bradycardia following stimulation of the carotid body chemoreceptors in the dog. J. Physiol..

[163] André S., Andreozzi F., Van Overstraeten C., Youssef S.B., Bold I., Carlier S., Gruwez A., Bruyneel A.V., Bruyneel M. (2020). Cardiometabolic comorbidities in obstructive sleep apnea patients are related to disease severity, nocturnal hypoxemia, and decreased sleep quality. Respir. Res..

[164] Gutierrez A., Van Wagoner D.R. (2015). Oxidant and inflammatory mechanisms and targeted therapy in atrial fibrillation: An update. J. Cardiovasc. Pharmacol..

[165] Sin D.D., Fitzgerald F., Parker J.D., Newton G., Floras J.S., Bradley T.D. (1999). Risk factors for central and obstructive sleep apnea in 450 men and women with congestive heart failure. Am. J. Respir. Crit. Care Med..

[166] Mariani S., Fiore D., Barbaro G., Basciani S., Saponara M., D’Arcangelo E., Ulisse S., Moretti C., Fabbri A., Gnessi L. (2013). Association of epicardial fat thickness with the severity of obstructive sleep apnea in obese patients. Int. J. Cardiol..

[167] Liak C., Fitzpatrick M. (2011). Coagulability in obstructive sleep apnea. Can. Respir. J..

[168] Healey J.S., Connolly S.J., Gold M.R., Israel C.W., Van Gelder I.C., Capucci A. (2012). Subclinical atrial fibrillation and the risk of stroke. N. Engl. J. Med..

[169] Rahangdale S., Yeh S.Y., Novack V., Stevenson K., Barnard M.R., Furman M.I., Frelinger A.L., Michelson A.D., Malhotra A. (2011). The influence of intermittent hypoxemia on platelet activation in obese patients with obstructive sleep apnea. J. Clin. Sleep Med..

[170] Jensen M.H., Dalgaard F., Laub R.R., Gottlieb V., Nielsen O.W., Hansen J., Hansen M.L., Jennum P., Lamberts M. (2023). Prevalence of sleep apnea in unselected patients with atrial fibrillation by a home-monitoring device: The DAN-APNO study. IJC Heart Vasc..

[171] Wessendorf T.E., Thilmann A.F., Wang Y.M., Schreiber A., Konietzko N., Teschler H. (2000). Fibrinogen levels and obstructive sleep apnea in ischemic stroke. Am. J. Respir. Crit. Care Med..

[172] Miyasaka Y., Barnes M.E., Petersen R.C., Cha S.S., Bailey K.R., Gersh B.J. (2007). Risk of dementia in stroke-free patients diagnosed with atrial fibrillation: Data from a community-based cohort. Eur. Heart J..

[173] Nakanishi K., Tajima F., Nakata Y., Osada H., Sugiyama K., Maruta H., Kawai T., Suzuki M., Torikata C. (1997). Hypercoagulable state in a hypobaric, hypoxic environment causes non-bacterial thrombotic endocarditis in rats. J. Pathol..

[174] Wattigney W.A., Mensah G.A., Croft J.B. (2003). Increasing trends in hospitalization for atrial fibrillation in the United States, 1985 through 1999: Implications for primary prevention. Circulation.

[175] Kelley N., Jeltema D., Duan Y., He Y. (2019). The NLRP3 inflammasome: An overview of mechanisms of activation and regulation. Int. J. Mol. Sci..

[176] Yao C., Veleva T., Scott L., Cao S., Li L., Chen G., Jeyabal P., Pan X., Alsina K.M., Abu-Taha I. (2018). Enhanced cardiomyocyte NLRP3 inflammasome signaling promotes atrial fibrillation. Circulation.

[177] Toldo S., Abbate A. (2018). The NLRP3 inflammasome in acute myocardial infarction. Nat. Rev. Cardiol..

[178] Lavie L. (2015). Oxidative stress in obstructive sleep apnea and intermittent hypoxia--revisited--the bad ugly and good: Implications to the heart and brain. Sleep Med. Rev..

[179] Gambardella J., Sorriento D., Ciccarelli M., Del Giudice C., Fiordelisi A., Napolitano L., Trimarco B., Iaccarino G., Santulli G. (2017). Functional Role of Mitochondria in Arrhythmogenesis. Adv. Exp. Med. Biol..

[180] Zhou T., Chuang C.C., Zuo L. (2015). Molecular characterization of reactive oxygen species in myocardial ischemia-reperfusion injury. BioMed Res. Int..

[181] Aon M.A., Cortassa S., O’Rourke B. (2008). Mitochondrial oscillations in physiology and pathophysiology. Adv. Exp. Med. Biol..

[182] Jelic S., Lederer D.J., Adams T., Padeletti M., Colombo P.C., Factor P.H., Le Jemtel T.H. (2010). Vascular inflammation in obesity and sleep apnea. Circulation.

[183] Drager L.F., Polotsky V.Y., Lorenzi-Filho G. (2011). Obstructive sleep apnea: An emerging risk factor for atherosclerosis. Chest.

[184] Childs B.G., Durik M., Baker D.J., van Deursen J.M. (2015). Cellular senescence in aging and age-related disease. Nat. Med..

[185] Donato A.J., Morgan R.G., Walker A.E., Lesniewski L.A. (2015). Cellular and molecular biology of aging endothelial cells. J. Mol. Cell Cardiol..

[186] Greco C.M., Condorelli G. (2015). Epigenetic modifications and noncoding RNAs in cardiac hypertrophy and failure. Nat. Rev. Cardiol..

[187] Condorelli G., Latronico M.V., Cavarretta E. (2014). microRNAs in cardiovascular diseases: Current knowledge and the road ahead. J. Am. Coll. Cardiol..

[188] Dawson K., Wakili R., Ordög B., Clauss S., Chen Y., Iwasaki Y., Voigt N., Qi X.Y., Sinner M.F., Dobrev D. (2013). MicroRNA29: A mechanistic contributor and potential biomarker in atrial fibrillation. Circulation.

[189] Santamaria-Martos F., Benítez I.D., Ortega F., Zapater A., Giron C., Pinilla L., Pascual L., Cortijo A., Dalmases M., Fernandez-Real J.M. (2019). Circulating microRNA profiles as potential biomarkers in obstructive sleep apnea. Sleep Med. Rev..

[190] Yuana Y., Sturk A., Nieuwland R. (2013). Extracellular vesicles in physiological and pathological conditions. Blood Rev..

[191] Sluijter J.P.G., Davidson S.M., Boulanger C.M., Buzás E.I., de Kleijn D.P.V., Engel F.B., Giricz Z., Hausenloy D.J., Kishore R., Lecour S. (2018). Extracellular vesicles in diagnostics and therapy of the ischemic heart: Position paper from the working group on cellular biology of the heart of the European Society of Cardiology. Cardiovasc. Res..

[192] Iacobellis G. (2022). Epicardial adipose tissue in contemporary cardiology. Nat. Rev. Cardiol..

[193] Mahajan R., Nelson A., Pathak R.K., Middeldorp M.E., Wong C.X., Twomey D.J., Carbone A., Teo K., Agbaedeng T., Linz D. (2018). Electroanatomical remodeling of the atria in obesity: Impact of adjacent epicardial fat. JACC Clin. Electrophysiol..

[194] Nalliah C.J., Sanders P., Kottkamp H., Kalman J.M. (2016). The role of obesity in atrial fibrillation. Eur. Heart J..

[195] Wong C.X., Abed H.S., Molaee P., Nelson A.J., Brooks A.G., Sharma G., Leong D.P., Lau D.H., Middeldorp M.E., Roberts-Thomson K.C. (2011). Pericardial fat is associated with atrial fibrillation severity and ablation outcome. J. Am. Coll. Cardiol..

[196] Lombardi C., Faini A., Mariani D., Gironi F., Castiglioni P., Parati G. (2020). Nocturnal arrhythmias and heart-rate swings in patients with obstructive sleep apnea syndrome treated with beta blockers. J. Am. Heart Assoc..

[197] Ucak S., Dissanayake H., Sutherland K., de Chazal P., Cistulli P. (2021). Heart rate variability and obstructive sleep apnea: Current perspectives and novel technologies. J. Sleep Res..

[198] Verrier R.L., Dickerson L.W. (1991). Autonomic nervous system and coronary blood flow changes related to emotional activation and sleep. Circulation.

[199] Kiuchi M.G., Nolde J.M., Villacorta H., Carnagarin R., Chan J.J.S.Y., Lugo-Gavidia L.M., Ho J.K., Matthews V.B., Dwivedi G., Schlaich M.P. (2019). New approaches in the management of sudden cardiac death in patients with heart failure—Targeting the sympathetic nervous system. Int. J. Mol. Sci..

[200] Linz D., Nattel S., Kalman J.M., Sanders P. (2021). Sleep apnea and atrial fibrillation. Card. Electrophysiol. Clin..

[201] Roden D.M., Iansmith D.H. (1987). Effects of low potassium or magnesium concentrations on isolated cardiac tissue. Am. J. Med..

[202] Goodfriend T.L. (2008). Obesity, sleep apnea, aldosterone, and hypertension. Curr. Hypertens. Rep..

[203] Berger K.I., Ayappa I., Sorkin I.B., Norman R.G., Rapoport D.M., Goldring R.M. (2000). CO_2_ homeostasis during periodic breathing in obstructive sleep apnea. J. Appl. Physiol..

[204] Joung B., Chen P.S. (2015). Function and dysfunction of human sinoatrial node. Korean Circ. J..

[205] Antzelevitch C., Burashnikov A. (2011). Overview of basic mechanisms of cardiac arrhythmia. Card. Electrophysiol. Clin..

[206] Padeletti M., Zacà V., Mondillo S., Jelic S. (2014). Sleep-disordered breathing increases the risk of arrhythmias. J. Cardiovasc. Med..

[207] Benjafield A.V., Ayas N.T., Eastwood P.R., Heinzer R., Ip M.S.M., Morrell M.J., Nunez C.M., Patel S.R., Penzel T., Pepin J.L. (2019). Estimation of the global prevalence and burden of obstructive sleep apnoea: A literature-based analysis. Lancet Respir. Med..

[208] Patel N., Donahue C., Shenoy A., Patel A., El-Sherif N. (2017). Obstructive sleep apnea and arrhythmia: A systemic review. Int. J. Cardiol..

[209] Gami A.S., Somers V.K. (2004). Obstructive sleep apnea, metabolic syndrome, and cardiovascular outcomes. Eur. Heart J..

[210] Linz D., McEvoy R.D., Cowie M.R., Somers V.K., Nattel S., Lévy P., Kalman J.M., Sanders P. (2018). Associations of obstructive sleep apnea with atrial fibrillation and continuous positive airway pressure treatment. JAMA Cardiol..

[211] Lubitz S.A., Yin X., Lin H., Kolek M., Smith J.G., Trompet S., Rienstra M., Rost N.S., Teixeira P.L., Almgren P. (2017). Genetic risk prediction of atrial fibrillation. Circulation.

[212] Tucker N.R., Ellinor P.T. (2014). Emerging directions in the genetics of atrial fibrillation. Circ. Res..

[213] Narkiewicz K., Somers V.K. (2003). Sympathetic nerve activity in obstructive sleep apnoea. Acta Physiol. Scand..

[214] Cremaschi chi R.C., Buitrago-Ricaute N., Novak P., Coelho F.M.S., Borges V. (2025). Autonomic Cardiovascular Assessment in Obstructive Sleep Apnea Using Beat-to-Beat Blood Pressure: A Pilot Study. Sleep Sci..

[215] Kottkamp H. (2013). Human atrial fibrillation substrate: Towards a specific fibrotic atrial cardiomyopathy. Eur. Heart J..

[216] Nattel S., Harada M. (2014). Atrial remodeling and atrial fibrillation: Recent advances and translational perspectives. J. Am. Coll. Cardiol..

[217] Marrouche N.F., Wilber D., Hindricks G., Jais P., Akoum N., Marchlinski F., Kholmovski E., Burgon N., Hu N., Mont L. (2014). Association of atrial tissue fibrosis identified by delayed enhancement MRI and atrial fibrillation catheter ablation: The DECAAF study. JAMA.

[218] Szymanski F.M., Filipiak K.J., Platek A.E., Hrynkiewicz-Szymanska A., Kotkowski M., Kozluk E., Kiliszek M., Sierdzinski J., Opolski G. (2015). Presence and severity of obstructive sleep apnea and remote outcomes of atrial fibrillation ablations—A long-term prospective, cross-sectional cohort study. Sleep Breath..

[219] Kendzerska T., Gershon A.S., Atzema C., Dorian P., Mangat I., Hawker G., Leung R.S. (2018). Sleep Apnea Increases the Risk of New Hospitalized Atrial Fibrillation: A Historical Cohort Study. Chest.

[220] Regitz-Zagrosek V., Kararigas G. (2017). Mechanistic pathways of sex differences in cardiovascular disease. Physiol. Rev..

[221] Won C.H.J., Reid M., Sofer T., Azarbarzin A., Purcell S., White D., Wellman A., Sands S., Redline S. (2020). Sex differences in obstructive sleep apnea phenotypes, the multi-ethnic study of atherosclerosis. Sleep.

[222] Linz D., Baumert M., Catcheside P., Floras J., Sanders P., Lévy P., Cowie M.R., Doug McEvoy R. (2018). Assessment and interpretation of sleep disordered breathing severity in cardiology: Clinical implications and perspectives. Int. J. Cardiol..

[223] Ryan S., Taylor C.T., McNicholas W.T. (2009). Systemic inflammation: A key factor in the pathogenesis of cardiovascular complications in obstructive sleep apnoea syndrome?. Thorax.

[224] Vgontzas A.N., Papanicolaou D.A., Bixler E.O., Hopper K., Lotsikas A., Lin H.M., Kales A., Chrousos G.P. (2000). Sleep apnea and daytime sleepiness and fatigue: Relation to visceral obesity, insulin resistance, and hypercytokinemia. J. Clin. Endocrinol. Metab..

[225] Ho J.E., Yin X., Levy D., Vasan R.S., Magnani J.W., Ellinor P.T., McManus D.D., Lubitz S.A., Larson M.G., Benjamin E.J. (2014). Galectin 3 and incident atrial fibrillation in the community. Am. Heart J..

[226] Januzzi J.L., Peacock W.F., Maisel A.S., Chae C.U., Jesse R.L., Baggish A.L., O’Donoghue M., Sakhuja R., Chen A.A., van Kimmenade R.R. (2007). Measurement of the interleukin family member ST2 in patients with acute dyspnea: Results from the PRIDE (Pro-brain natriuretic peptide investigation of dyspnea in the Emergency Department) study. J. Am. Coll. Cardiol..

[227] Prasad A., Bekker P., Tsimikas S. (2012). Advanced glycation end products and diabetic cardiovascular disease. Cardiol. Rev..

[228] Shaffer F., Ginsberg J.P. (2017). An overview of heart rate variability metrics and norms. Front. Public Health.

[229] Narkiewicz K., Montano N., Cogliati C., van de Borne P.J., Dyken M.E., Somers V.K. (1998). Altered cardiovascular variability in obstructive sleep apnea. Circulation.

[230] Somers V.K., White D.P., Amin R., Abraham W.T., Costa F., Culebras A., Daniels S., Floras J.S., Hunt C.E., Olson L.J. (2008). Sleep Apnea and Cardiovascular Disease: An American Heart Association/American College of Cardiology Foundation Scientific Statement from the American Heart Association Council for High Blood Pressure Research Professional Education Committee, Council on Clinical Cardiology, Stroke Council, and Council on Cardiovascular Nursing. Circulation.

[231] Pathan F., D’Elia N., Nolan M.T., Marwick T.H., Negishi K. (2017). Normal ranges of left atrial strain by speckle-tracking echocardiography. J. Am. Soc. Echocardiogr..

[232] Cameli M., Lisi M., Focardi M., Reccia R., Natali B.M., Sparla S., Mondillo S. (2012). Left atrial deformation analysis by speckle tracking echocardiography for prediction of cardiovascular outcomes. Am. J. Cardiol..

[233] Huang B., Liu H., Scherlag B.J., Sun L., Xing S., Xu J., Luo M., Guo Y., Cao G., Jiang H. (2021). Atrial fibrillation in obstructive sleep apnea: Neural mechanisms and emerging therapies. Trends Cardiovasc. Med..

[234] Birză M.R., Negru A.G., Frent Ș.M., Florescu A.-R., Popa A.M., Manzur A.R., Lascu A., Mihaicuța S. (2025). New insights of cardiac arrhythmias associated with sleep-disordered breathing: From mechanisms to clinical implications—A narrative review. J. Clin. Med..

[235] DiCaro M.V., Lei K., Yee B., Tak T. (2024). The effects of obstructive sleep apnea on the cardiovascular system: A comprehensive review. J. Clin. Med..

[236] Sousa S., Drummond M., Bugalho A. (2025). Prevalence and diagnosis of obstructive sleep apnea in atrial fibrillation patients: A systematic review. J. Clin. Med..

[237] Monahan K., Brewster J., Wang L., Parvez B., Goyal S., Roden D.M., Darbar D. (2012). Relation of the severity of obstructive sleep apnea in response to anti-arrhythmic drugs in patients with atrial fibrillation or atrial flutter. Am. J. Cardiol..

[238] Koscova Z., Rad A.B., Nasiri S., Reyna M.A., Sameni R., Trotti L.M., Sun H., Turley N., Stone K.L., Thomas R.J. (2024). From sleep patterns to heart rhythm: Predicting atrial fibrillation from overnight polysomnograms. J. Electrocardiol..

[239] Li W., Yan S., Zhao J., Ding X., Zhang S., Wang D., Liu L., Peng W., Li H., Wang D. (2015). Metoprolol Inhibits cardiac apoptosis and fibrosis in a canine model of chronic obstructive sleep apnea. Cell. Physiol. Biochem..

[240] Dai H., Yuan Y., Yin S., Zhang Y., Han Y., Sun L., Li T., Xu J., Sheng L., Gong Y. (2019). Metoprolol inhibits profibrotic remodeling of epicardial adipose tissue in a canine model of chronic obstructive sleep apnea. J. Am. Heart Assoc..

[241] Sun L., Yan S., Wang X., Zhao S., Li H., Wang Y., Lu S., Dong X., Zhao J., Yu S. (2017). Metoprolol prevents chronic obstructive sleep apnea-induced atrial fibrillation by inhibiting structural, sympathetic nervous and metabolic remodeling of the atria. Sci. Rep..

[242] Takahashi K., Ueda S., Kobayashi T., Nishiyama A., Fujisawa Y., Sugaya T., Shiota S., Takahashi K., Gohda T., Horikoshi S. (2018). Chronic intermittent hypoxia-mediated renal sympathetic nerve activation in hypertension and cardiovascular disease. Sci. Rep..

[243] Tavares L., Rodríguez-Mañero M., Kreidieh B., Ibarra-Cortez S.H., Chen J., Wang S., Markovits J., Barrios R., Valderrábano M. (2019). Cardiac afferent denervation abolishes ganglionated plexi and sympathetic responses to apnea. Circ. Arrhythmia Electrophysiol..

[244] Stavrakis S., Humphrey M.B., Scherlag B.J., Hu Y., Jackman W.M., Nakagawa H., Lockwood D., Lazzara R., Po S.S. (2015). Low-level transcutaneous electrical vagus nerve stimulation suppresses atrial fibrillation. J. Am. Coll. Cardiol..

[245] Li T., Rong L., Gao Y., Cheng W. (2024). The causal relationship between obesity, obstructive sleep apnea and atrial fibrillation: A study based on mediated Mendelian randomization. Front. Cardiovasc. Med..

[246] Fein A.S., Shvilkin A., Shah D., Haffajee C.I., Das S., Kumar K., Kramer D.B., Zimetbaum P.J., Buxton A.E., Josephson M.E. (2013). Treatment of obstructive sleep apnea reduces the risk of atrial fibrillation recurrence after catheter ablation. J. Am. Coll. Cardiol..

